# Identification of Eph receptor signaling as a regulator of autophagy and a therapeutic target in colorectal carcinoma

**DOI:** 10.1002/1878-0261.12576

**Published:** 2019-10-23

**Authors:** Michael DiPrima, Dunrui Wang, Alix Tröster, Dragan Maric, Nekane Terrades‐Garcia, Taekyu Ha, Hyeongil Kwak, David Sanchez‐Martin, Denis Kudlinzki, Harald Schwalbe, Giovanna Tosato

**Affiliations:** ^1^ Laboratory of Cellular Oncology Center for Cancer Research (CCR) National Cancer Institute (NCI) Bethesda MD USA; ^2^ Center for Biomolecular Magnetic Resonance Institute for Organic Chemistry and Chemical Biology Johann Wolfgang Goethe‐University Frankfurt am Main Germany; ^3^ National Institutes of Neurological Disorders and Stroke National Institutes of Health (NIH) Bethesda MD USA; ^4^ Vasculitis Research Unit Department of Autoimmune Diseases Hospital Clinic University of Barcelona Spain

**Keywords:** cell death, colorectal cancer, Eph receptors, Ephrin ligand, tyrosine kinase signaling

## Abstract

Advanced colorectal carcinoma is currently incurable, and new therapies are urgently needed. We report that phosphotyrosine‐dependent Eph receptor signaling sustains colorectal carcinoma cell survival, thereby uncovering a survival pathway active in colorectal carcinoma cells. We find that genetic and biochemical inhibition of Eph tyrosine kinase activity or depletion of the Eph ligand EphrinB2 reproducibly induces colorectal carcinoma cell death by autophagy. Spautin and 3‐methyladenine, inhibitors of early steps in the autophagic pathway, significantly reduce autophagy‐mediated cell death that follows inhibition of phosphotyrosine‐dependent Eph signaling in colorectal cancer cells. A small‐molecule inhibitor of the Eph kinase, NVP‐BHG712 or its regioisomer NVP‐Iso, reduces human colorectal cancer cell growth *in vitro* and tumor growth in mice. Colorectal cancers express the EphrinB ligand and its Eph receptors at significantly higher levels than numerous other cancer types, supporting Eph signaling inhibition as a potential new strategy for the broad treatment of colorectal carcinoma.

**Abbreviations**
3‐MA: 3‐methyladenine
ATG: autophagy‐related gene
CMS: consensus molecular subtype
CPTAC: Clinical Proteome Tumor Analysis Consortium
HUVEC: human umbilical vein endothelial cells
KD: kinase‐deficient
LC3: light‐chain 3
LC3: microtubule‐associated protein‐1 light‐chain 3
NSCLC: non‐small‐cell lung cancer
RTK: receptor tyrosine kinase
TCGA: The Cancer Genome Atlas
TKI: tyrosine kinase inhibitor
WT: wild‐type

## 1 Introduction

Despite reduced incidence from screening, polypectomy, and other interventions, colorectal cancer remains a leading cause of cancer death (Welch and Robertson, 2016). The mutational process linked to colorectal carcinogenesis introduces genetic alterations that often result in functional loss of the tumor suppressors APC, TP53, and SMAD4 in conjunction with activation of the oncogene KRAS (Fearon and Vogelstein, 1990). Lineage‐tracing experiments have shown that the leucine‐rich repeat‐containing G‐protein‐coupled receptor‐5‐positive (Lgr5^+^) intestinal stem cells, which normally reside at the bottom of the intestinal crypt, are the cells of origin of intestinal adenomas, the precursors of colorectal cancer (Barker *et al*., 2007; Schepers *et al*., 2012). Most colorectal cancers contain Lgr5^+^ cells (Junttila *et al*., 2015; Shimokawa *et al*., 2017), which can produce both Lgr5^+^ and Lgr5^−^ cells, indicative of their self‐renewal and differentiation potential (Shimokawa *et al*., 2017).

Depletion of Lgr5^+^ cells from colorectal carcinomas reduced tumor growth in mice, but did not lead to tumor regression (Shimokawa *et al*., 2017; de Sousa e Melo *et al*., 2017), and this phenotype coincided with the more differentiated Lgr5^−^ cancer cells regaining their proliferative capacity and Lgr5^+^ status (Shimokawa *et al*., 2017; de Sousa e Melo *et al*., 2017). This plasticity mirrors that of the intestinal epithelium, where committed enterocytes and secretory cells can revert to becoming multipotent Lgr5^+^ stem cells in response to supportive niche signals at the base of the crypt (van Es *et al*., 2012; Tian *et al*., 2011). Since cell location contributes to the maintenance of Lgr5^+^ stem cell identity (Batlle and Clevers, 2017; Shoshkes‐Carmel *et al*., 2018), determinants of cell location may help identify new therapeutic targets for colorectal carcinoma.

B‐type Ephrin ligands and their Eph tyrosine kinase receptors comprise a family of transmembrane proteins that play pivotal roles in cell‐to‐cell communication (Kania and Klein, 2016). In the intestinal crypt, EphrinBs and EphBs regulate the normal crypt architecture and mark the position of stem cells and more mature enterocytes along the crypt/villous axis (Batlle *et al*., 2002; Holmberg *et al*., 2006; Kania and Klein, 2016). EphBs have also been shown to promote the proliferation of intestinal progenitor cells (Holmberg *et al*., 2006).

In experimental models of intestinal tumorigenesis, EphB receptors promoted the development of intestinal adenomas and inhibited the transition of adenomas into invasive carcinomas (Batlle *et al*., 2005; Cortina *et al*., 2007; Genander *et al*., 2009; Kundu *et al*., 2015). In human colorectal carcinogenesis, the role of B‐type ephrins and Ephs is currently unclear. Correlative studies suggested that EphB suppresses colorectal cancer progression (Batlle *et al*., 2005) and that mutant EphB increases colorectal carcinoma metastasis (Mathot *et al*., 2017). Other studies suggested that EphB promotes colorectal carcinoma growth augmenting cell replication, motility, and tumor vascularization (Kadife *et al*., 2018; Lv *et al*., 2016; McCall *et al*., 2016). Here, we show that phosphotyrosine‐dependent Eph signaling controls colorectal carcinoma cell survival.

## 2 Materials and methods

### 2.1 Cells, cell culture, and materials

HT‐29 (ATCC, Manassas, VA, USA; HTB‐38) and HCT 116 (ATCC; CCL‐247) cell lines were grown in McCoy5A medium (Corning, Corning, NY, USA; 10‐050‐CV) supplemented with 10% FBS (Sigma‐Aldrich, St. Louis, MO, USA; F2442) and penicillin/streptomycin (ThermoFisher Scientific, Waltham, MA, USA; 15140122); DLD‐1 (ATCC; CCL‐221), Colo 205 (ATCC; CCL‐222), and H747 (ATCC; CCL‐252) cell lines were grown in RPMI‐1640 medium (Corning; 10‐040‐CV), supplemented with 10% FBS and penicillin/streptomycin; SW620 (ATCC; CCL‐227), SW480 (ATCC; CCL‐228), RKO (ATCC; CCL‐2577), SK‐Co‐1 (ATCC; HTB‐39), MG‐63 (ATCC; CRL‐1427), MCF‐7 (ATCC; HTB‐22), A431 (ATCC; CRL‐1555), and MDA‐MB‐068 (ATCC; HTB‐26) cell lines were grown in DMEM (Corning; 10‐017‐CV), supplemented with 10% FBS and penicillin/streptomycin. LoVo (ATCC; CCL‐229) and Sum149 (BioIVT, Detroit, MI, USA) cell lines were grown in F12K medium (ATCC; 30‐2004) supplemented with 10% FBS and penicillin/streptomycin. Human umbilical vein endothelial cells (HUVEC) from an individual donor were purchased from Lifeline Cell Technologies (Frederick, MD, USA; FC‐0003); human pooled neonatal dermal microvascular endothelial cells (hDMEC) were purchased from Lonza Biosciences (Basel, Switzerland; CC‐2516). HUVEC and hDMEC were cultured in M199 (Corning; 10‐060‐CV) medium supplemented with 50 μg·mL^−1^ ascorbic acid (Sigma; A4034), 25 μg·mL^−1^ heparin (Sigma; H3149), 2 mm l‐glutamine (ThermoFisher; 25030081), penicillin/streptomycin, endothelial growth factor supplement (Sigma; E9640), 5% human serum (Sigma; 4522), 20% heat‐inactivated newborn calf serum (Sigma; N4637). All lines were used for a maximum number of 10–15 passages over ~ 4 years. All cell lines tested negative for mycoplasma (yearly) and MTBM (Molecular Testing of Biological Materials; tested once). NVP‐BHG712 (Troster *et al*., 2018), NVP‐Iso (Sigma; SML0333, Lots 042M4735V and 124M4703V), spautin‐1 (Sigma; SML0440), rapamycin (Sigma; R8781), and Z‐VAD‐FMK (R&D Systems, Minneapolis, MN, USA; FMK001) were reconstituted in DMSO. Diluent control (DMSO in culture medium) was adjusted to the DMSO concentration of the drug. Chloroquine (Sigma; C6628) was reconstituted in PBS. 3‐Methyladenine (3‐MA; Selleckchem, Nepean, ON, Canada; S2767) was dissolved into culture medium and used immediately.

### 2.2 Gene expression and silencing

RNA isolated from homogenized cells (Qiashredder, Qiagen, Holden, Germany; 79656) using RNEasy kit (Qiagen; 74106) was then used to synthesize cDNA with QuantiTect Reverse Transcription Kit (Qiagen; 205311) according to the manufacturer's recommendations. mRNA expression was measured by real‐time PCR on ABI 7900 thermocycler (Applied Biosystems, Foster City, CA, USA; 60 °C annealing temperature, 40 amplification cycles) using FastStart Universal SYBR Green Master Mix (ROX; Roche, Basel, Switzerland; 04913914001). Ct (cycle threshold) values were calculated by sds 2.4.1 software (Applied Biosystems). Primers are listed in Table S1. Lentiviral shRNA particles for targeting human EphrinB2, EphB2, and EphB4 (Mission shRNA vectors; Sigma), and for expression of pGK empty vector (Sigma; SCH001), nontargeting shRNA (Sigma; SCH002), LC3 (pk‐LC3; Addgene, Cambridge, MA, USA), wild‐type human EphB4, and kinase‐deficient EphB4 mutant (K647R/KD) (Yang *et al*., 2006) were produced in 293T cells with third‐generation lentiviral packaging system (pMDLg/pRRE; pRSV‐Rev, and VSVG envelop plasmid), as described (Kwak *et al*., 2016; Salvucci *et al*., 2015). Lentiviral vectors are listed in Table S2. For siRNA‐mediated silencing, cells were transfected with ON‐Target PLUS nontargeting pool siRNA (Dharmacon, Lafayette, CO, USA; D‐001810‐10‐05) or ON‐Target PLUS siATG5 SMARTpool (Dharmacon; L‐004374‐00‐0005) using Lipofectamine RNAiMAX (ThermoFisher; 13778030) according to the manufacturer's recommendations.

### 2.3 Immunoprecipitation, immunoblotting, and measurement of phospho‐EphB4

Lysates from cells, harvested from dishes by scraping with ice‐cold PBS, were suspended in freshly prepared TNTG lysis buffer [1× TBS (Quality Biological, Gaithersburg, MD, USA; 351‐086‐101), 1% Triton X‐100 (Sigma; T9284), 20% glycerol (ThermoFisher; 17904), 1× protease inhibitor cocktail (ThermoFisher; 78425), and 1× phosphatase inhibitor (Sigma; P5726)]. After incubation (1 h at 4 °C), lysates were spun (10 000 ***g***, 20 min) and supernatants stored at −20 °C.

For immunoprecipitation, cell lysates (250 μg) precleared with 25 μL protein G DynaBeads (ThermoFisher; 10004D) for 30 min at 4 °C rotating, were incubated with 1 μg immunoprecipitating antibody (Table S3) overnight at 4 °C rotating. After incubation, 25 μL of protein G DynaBeads was added to the antibody/lysate complex for 1 h at 4 °C rotating. The beads/antibody/protein complex was washed twice with TNTG buffer, twice with HNTG high salt buffer (20 mm HEPES, 500 mm NaCl, Quality Biological; 351‐036‐101, 1% Triton X‐100, 10% glycerol), twice with HNTG low salt (20 mm HEPES, 150 mm NaCl, 1% Triton X‐100, 10% glycerol). Protein was eluted from the beads by incubation with sample buffer (1× LDS; ThermoFisher; NP0007) containing 5% beta‐mercaptoethanol (Sigma; M3148) at 100 °C for 5 min.

Protein lysates and immunoprecipitates were separated by SDS/PAGE using NuPage 4–12% Bis–Tris gels (ThermoFisher; NP0321, NP0323), with either MOPS (ThermoFisher; NP0001) or MES (ThermoFisher; NP0002) running buffer. Separated proteins were transferred to a nitrocellulose membrane using the iBlot dry transfer system (ThermoFisher; IB1001). Membranes were blocked in TBS supplemented with 0.05% Tween‐20 (Sigma; P1379) and 5% nonfat dry milk (Lab Scientific, Highlands, NJ, USA; M0841) for at least 1 h at room temperature. Membranes were incubated overnight at 4 °C with primary antibody at appropriate dilutions (Table S3). After washing with TBS‐T (TBS with 0.05% Tween 20), membranes were incubated with the appropriate HRP (horseradish peroxidase)‐linked secondary antibody, IgG sheep anti‐mouse IgG (NA931), IgG donkey anti‐rabbit IgG (NA934) (both from GE LifeSciences, Marlborough, MA, USA), or IgG rabbit anti‐goat (ThermoFisher; A27014). Blots were developed using ECL prime (GE LifeSciences; RPN2232) and digitally captured on an LAS4000 (GE LifeSciences). Western blot bands were quantified using fiji (Schindelin *et al*., 2012).

Human phosphotyrosine EphB4 content was measured by ELISA (R&D Systems; DYC4057) in tumor lysates prepared with TissueLyser LT (Qiagen; 85600) in TNTG lysis buffer, according to the manufacturer's recommendations. Total EphB4 content in the tumor lysates was measured by immunoblotting with human EphB4‐specific antibody (Table S3).

### 2.4 Cell proliferation, cell cycle, and cell death

Cell proliferation was measured by ^3^H‐thymidine incorporation. Briefly, 0.5 μCi ^3^H‐thymidine (Perkin Elmer, Waltham, MA, USA; NET027WW001MC) was added to cells in 200 μL of culture medium in a 96‐well plate for 8–18 h. Plates were frozen to stop cell growth, cells harvested onto glass fiber filters (Perkin Elmer; 1450‐421), dried by microwave, wet with FiltronX liquid scintillation fluid (National Diagnostics, Atlanta, GA, USA; LS‐201), and incorporated radioactivity counted in a liquid scintillation counter (Perkin Elmer; MicroBeta‐1450 or MicroBeta‐2450).

Monolayer cell confluency over time was measured by IncuCyte HD (Essen BioSciences, Ann Arbor, MI, USA) imaging. Specifically, cells growing as monolayers were photographed every 6 h for 1 week with a 20× objective; cells were allowed to attach for at least 18 h prior to imaging; 16 images were taken/per well/per time point. Cell viability was measured by flow cytometry, as described (Salvucci *et al*., 2015). Briefly, floating and adherent cells were collected by centrifugation, pelleted, and suspended in PBS−/− (ThermoFisher; 10010‐049) supplemented with 1% BSA (Sigma; A2153), 5 mm EDTA (Quality Biological; 351‐027), 10 mm glucose (Sigma; D9434), and 10 mm HEPES (Corning; 25‐060‐CI). Cells (1 × 10^6^ cells·mL^−1^) were stained with 50 μg Hoechst 33342 (ThermoFisher; H3570) or 25 μm DRAQ5 (BioLegend, San Diego, CA, USA; 424101) for 60 min and 2 μg propidium iodide (ThermoFisher; P3566) for 15 min; analysis was performed on a MoFlo Astrios EQ flow cytometer (Beckman Coulter, Brea, CA, USA; B25982) or FACSCantoII (BD Biosciences, Franklin Lakes, NJ, USA; 338962). Results were analyzed by flojo software (FlowJo LLC, Ashland, OR, USA). Cell cycle was assessed by flow cytometry. Adherent cells were harvested, washed with PBS, and fixed for 30 min in cold 70% ethanol at 4 °C. Cells were washed twice with PBS, treated with 2.5 μg·mL^−1^ RNaseA (ThermoFisher; 12091039) for 30 min at room temperature, and then stained with 10 μg propidium iodide for 15 min prior to analysis performed on FACS CantoII and analyzed by flojo (cell cycle tool).

### 2.5 EphrinB2‐Fc cell stimulation

This protocol was adapted from a previous description (Bochenek *et al*., 2010). Briefly, cells were plated at 50% confluency 24 h prior to stimulation. Human EphrinB2‐Fc (R&D Systems; 7397‐EB‐050) and human IgG‐Fc (JacksonImmuno Research, West Grove, PA, USA; 009‐000‐008) were individually clustered with anti‐human IgG‐Fc‐specific antibody (ThermoFisher; 628400) for 1 h at 37 °C by incubating individually EphrinB2‐Fc and human IgG‐Fc (200 μg·mL^−1^) with anti‐human IgG‐Fc (200 μg·mL^−1^). Clustered EphrinB2‐Fc and control IgG‐Fc (1 μg·mL^−1^ clustered EphrinB2‐Fc final concentration) was used to stimulate cells for 15 min at 37 °C. To help preserve protein phosphorylation, cells were treated with 100 μm pervanadate for 15 min at 37 °C prior to preparation of cell lysates. Pervanadate was generated by combining 100 mm sodium orthovanadate (Sigma; S6508) with 3% hydrogen peroxide (Sigma; 216763) for 30 min at 37 °C.

### 2.6 Immunostaining and microscopy

Tumor sections from 4% PFA‐fixed OCT‐embedded tissues were processed for histology and immunostained as we previously described (Kwak *et al*., 2016; Salvucci *et al*., 2015). After thawing, sections were incubated with Uni‐Trieve solution (Innovex Biosciences, Richmond, CA, USA; NB325) at 75 °C for 45 min. After washing three times with wash buffer (PBS + 1% Triton X‐100), blocking [10% glycerol, 5% donkey serum (Sigma; D9663), TBS, 0.4% Triton X‐100] for 1 h at room temperature, washing once with wash buffer, tissues were incubated overnight at 4 °C with primary antibody diluted in buffer (TBS, 10% glycerol, 0.5% BSA, 0.4% Triton X‐100). Slides were then washed three times with wash buffer and incubated with secondary antibodies [Alexa Fluor 488 donkey anti‐rat IgG (ThermoFisher; A21208) and Alexa Fluor 594 donkey anti‐rabbit IgG (ThermoFisher; A21207)] for 1 h at 4 °C. The slides were washed three times with wash buffer, fixed with 4% PFA/PBS for 15 min at room temperature, washed once with wash buffer, and mounted with DAPI‐containing mounting medium (Southern Biotech, Birmingham, AL, USA; 0100‐20). Antibodies are listed in Table S3. Sections were imaged by confocal microscopy (780 confocal microscope; Carl Zeiss, Oberkochen, Germany). Maximum intensity projections (zen Software 2.3 Blue Edition; Carl Zeiss) were used for quantification using cellprofiler software (Kamentsky *et al*., 2011) with automatic thresholding. Integrated intensity of CD31 was measured. The percent Ki‐67‐positive cells was measured by calculating the number of Ki‐67‐positive cells/total number DAPI^+^ nuclei. The area of cleaved caspase‐3‐positive area/total area was calculated by cellprofiler with automatic threshold. For LC3 quantification, cells stably expressing fluorescent pK (pHluorin‐mKate2) LC3 after lentiviral infection with pk‐LC were grown on autoclaved glass coverslips (ThermoFisher; 12‐545‐81) until 50% confluent. Coverslips were washed with PBS−/−, fixed with 4% PFA for 15 min at room temperature, washed with TBS, mounted with DAPI‐containing mounting media, allowed to harden overnight and sealed. Coverslips were imaged on a confocal Zeiss 780 microscope or an Olympus IX51 (Shinjuku, Tokyo, Japan) inverted microscope. Images were analyzed by cellprofiler using automatic thresholding to calculate the number of nuclei (DAPI staining) and the number of puncta. The results are expressed as the average number of puncta/cell or as percent cells with two or more puncta. Images were colorized with fiji software.

### 2.7 Tumor model

All animal studies were approved by the Institutional Animal Care and Use Committee of the CCR, National Cancer Institute (NCI), NIH. The studies were conducted in adherence to the *NIH Guide for the Care and Use of Laboratory Animals* (National Academies Press, 2011).

Female Nu/Nu mice (6–10‐week old, Charles River Laboratories) bearing subcutaneous tumors with an average tumor volume of 100 mm^3^ (*V* = *D*(*d* ^2^)/2, where (*D*) and (*d*) are, respectively, the longest and shortest perpendicular dimensions) were randomized to receive drug (NVP‐Iso 15 mg·kg^−1^) or diluent control as daily intraperitoneal injections (0.1 mL). Mice were dosed daily intraperitoneally (i.p.) with either formulation buffer (DMSO : PBS, 150 : 1150) or NVP‐Iso (15 mg·kg^−1^ diluted in formulation buffer). Total injection volume was 100 μL. The mouse diet was supplemented with gel meal (DietGel Boost, ClearH2O, Westbrook, ME, USA) during dosing. Mice were euthanized when any tumor reached a size of 20 mm in any direction. Tumors were removed in toto from the mice and tumor weight measured.

### 2.8 Data analysis and statistics

An unpaired 2‐tailed Student's *t‐*test was used for statistical analysis of differences between two groups (GraphPad Prism, La Jolla, CA, USA). *P*‐values < 0.05 were considered statistically significant. A 2‐tailed Mann–Whitney *U*‐test (GraphPad Prism) was used to calculate the statistical significance of differences in mRNA expression levels. *U*‐values < 0.05 were considered statistically significant. Mantel–Cox log‐rank test (GraphPad Prism) was used for analysis of statistical significance of differences in probability of patient survival by single gene expression levels. *P*‐values < 0.05 were considered statistically significant.

TCGA RNAseq (level three RNASseqV2) data set for colorectal carcinoma was compared to the combined data sets for stomach, breast, lung adenocarcinoma, lung squamous cell, hepatocellular carcinoma, ovarian, melanoma, uterine, and prostate cancers. Level three RNASseqV2 values were used to calculate the mean RNA values for each gene in each data set (colorectal carcinoma and other cancers combined). Statistical significance of group differences was calculated by two‐tailed Mann–Whitney *U*‐test (GraphPad Prism). *U*‐values < 0.05 were considered statistically significant.

EGEOD‐17538 (Smith *et al*., 2010) includes normalized gene expression data from 232 colorectal carcinoma specimens acquired by microarray analysis (U133 Plus 2.0 Array). For analysis of survival probability as a function of gene expression levels, gene expression data for individual genes were distinguished into ‘high expression’ and ‘low expression’ based on being above or below the group median. Survival probability was determined from Kaplan–Meier curves generated from the high and low expression groups, and the statistical significance of group differences was calculated using Mantel–Cox log‐rank test (GraphPad Prism software). *P*‐values < 0.05 were considered statistically significant.

Proteomics data (mzML files; Cancer Proteome Confirmatory Colon Study) were directly downloaded from the Clinical Proteome Tumor Analysis Consortium (CPTAC) data portal (https://cptac-data-portal.georgetown.edu/cptac/s/S037). MSfragger, a peptide identification tool (command line version), was used to determine protein abundance (Kong *et al*., 2017). Data are expressed as mean ± SEM; *P*‐values < 0.05 were considered statistically significant.

## 3 Results

### 3.1 Expression of EphrinB2 ligand and its receptors in colorectal carcinoma correlates with patient survival

**Figure 1:**
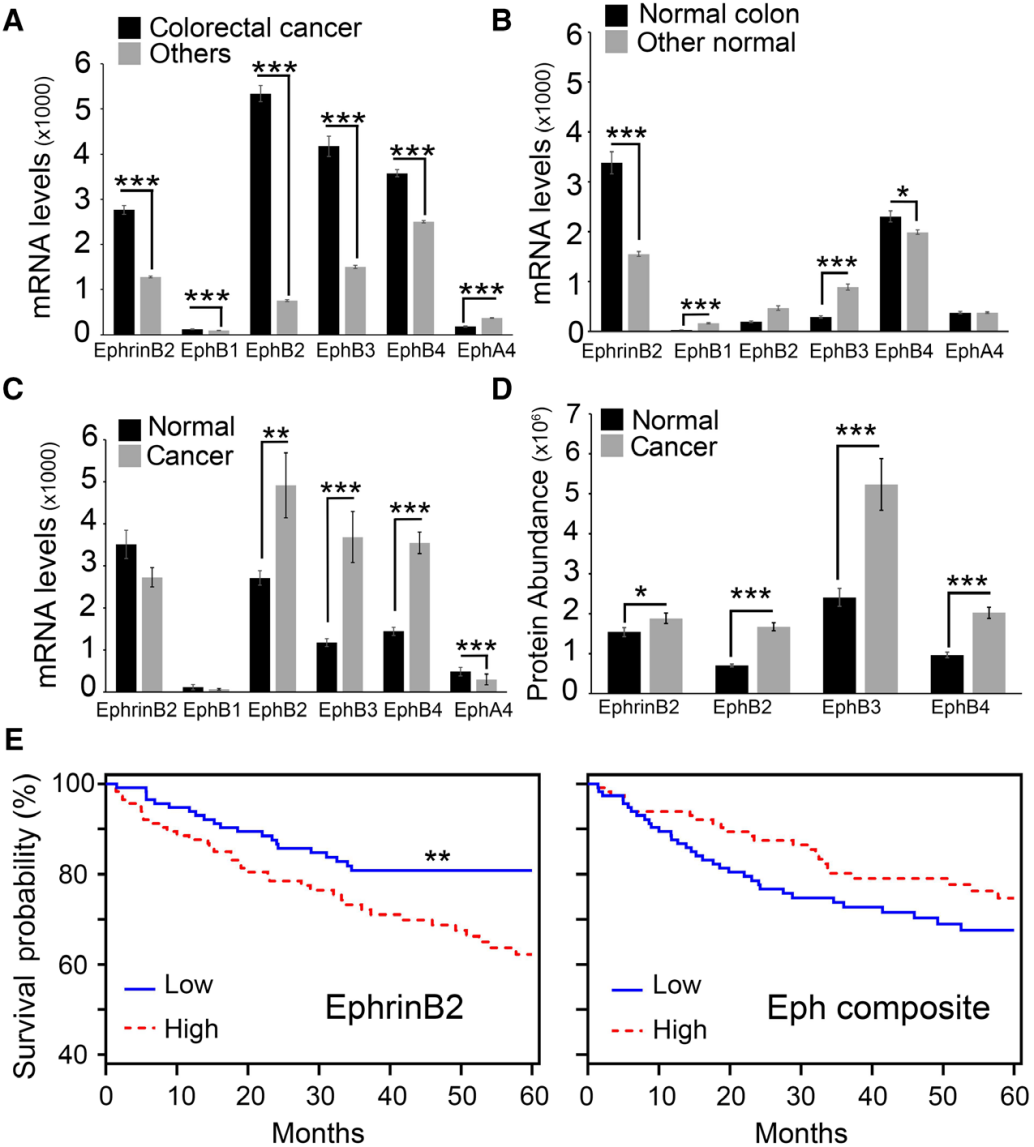
EphrinB2 and Eph expression in colorectal cancer. (A–C) mRNA expression of EphrinB2 ligand and Eph receptors in colorectal cancer (*n* = 269) and in other cancers (*n* = 3345) (A); in normal colon tissue (*n* = 41) and in other normal tissues (*n* = 346) (B); and in paired samples of tumor and normal colon tissue (*n* = 26) by RNAseq (C). All data are shown as mean (±SEM). Statistical significance of group differences by two‐tailed Mann–Whitney *U*‐test. (D) Protein abundance of EphrinB2 and its receptors EphB2, EphB3, and EphB4 in colon cancer (*n* = 100) and normal colon (*n* = 100) by mass spectrometry. Protein abundance is shown as mean (±SEM). Statistical significance of group differences by two‐tailed Mann–Whitney *U*‐test. (E) Relationship between high (above the median, *n* = 116) or low (below the median, *n* = 116) EphrinB2 or Eph receptors mRNA expression levels and survival probability at 60 months from diagnosis. Individual (EphrinB2) and composite (EphB1, EphB2, EphB3, EphB4, and EphA4) median high and low mRNA expression levels were calculated. Statistical significance of group differences from Kaplan–Meier probability estimates by log‐rank, Mantel–Cox test *P* values. *P < 0.05, **P ≤ 0.01 and ***P ≤ 0.001.

We examined the expression of EphrinB2 ligand and the EphrinB2 signaling receptors (EphB1, EphB2, EphB3, EphB4, and EphA4) (Kania and Klein, 2016; Noberini *et al*., 2012b) in colorectal carcinoma. Based on the RNA sequencing results from TCGA (The Cancer Genome Atlas) database (http://cancergenome.nih.gov), we determined that colorectal cancer tissues (*n* = 269) express significantly higher mRNA levels of EphrinB2, EphB1, EphB2, EphB3, and EphB4 than all other cancers surveyed (*n* = 3345; stomach *n* = 413; breast *n* = 901; lung adenocarcinoma *n* = 465; lung squamous cell carcinoma *n* = 407; hepatocellular carcinoma *n* = 132; ovarian *n* = 253; melanoma *n* = 82; uterine *n* = 365 and prostate *n* = 327), but lower levels of EphA4 (Fig. 1A). Additional analysis of TCGA data showed that normal colon tissues (*n* = 41) express similar or significantly lower mRNA levels of the receptors EphB1, EphB2, EphB3, and EphA4 compared to other normal tissues (*n* = 346; stomach *n* = 35; breast *n* = 92; lung *n* = 108; liver *n* = 50; melanoma *n* = 1; uterus *n* = 11; and prostate *n* = 49), but EphrinB2 and EphB4 mRNA levels are significantly higher (Fig. 1B). Also, analysis of paired normal colon tissue and colon cancer (*n* = 26) showed that mRNA levels of EphB2, EphB3, EphB4, and EphA4 are significantly lower in the normal tissue than in the adjacent cancer (Fig. 1C), whereas levels of EphrinB2 and EphB1 are similar (Fig. 1C).

The Clinical Proteomic Tumor Analysis Consortium (CPTAC)‐2 (https://proteomics.cancer.gov/data-portal) results mirrored the results of transcriptome analysis in showing that EphB2, EphB3, and EphB4 proteins are significantly more abundant in colon cancer compared to the adjacent normal colon tissue (*n* = 100; EphB1 and EphA4 proteins were not reported, presumably attributable to no detection). EphrinB2 protein levels were also modestly, although significantly increased in colon cancer compared to the normal adjacent tissue (*n* = 100) (Fig. 1D).

We looked at the relationship between patient survival probability and expression levels of EphrinB2 and its receptors in colorectal tumors (E‐GEOD‐17538: https://www.ncbi.nlm.nih.gov/geo/query/acc.cgi?acc=GSE317538). The probability of patient survival is significantly higher when EphrinB2 mRNA is expressed at low (below the group median) as opposed to high (above the group median) levels (Fig. 1E, left). To evaluate the impact of high or low receptor expression in colorectal cancer patients, we reasoned that all the EphrinB2 receptors can signal in response to EphrinB2 binding and are therefore functionally linked. Given that each colorectal cancer in this database expresses all the receptors, albeit at different levels, we examined the combined impact of all receptors on patient survival probability. We found that the probability of patient survival is similar when the expression of the five receptors (EphB1, EphB2, EphB3, EphB4, and EphA4) is low (below the group median) or high (above the group median) (Fig. 1E, right).

Overall, these results indicate that colorectal cancer is distinct from other cancer types in displaying higher expression of EphrinB2 and its receptors EphB1, EphB2, EphB3, and EphB4. Moreover, these results show that high expression of EphrinB2 in colorectal cancer is associated with a reduced probability of long‐term survival, suggesting a possible role of this protein in colorectal carcinoma.

### 3.2 EphrinB2 depletion reduces the viability of colorectal carcinoma cells

**Figure 2:**
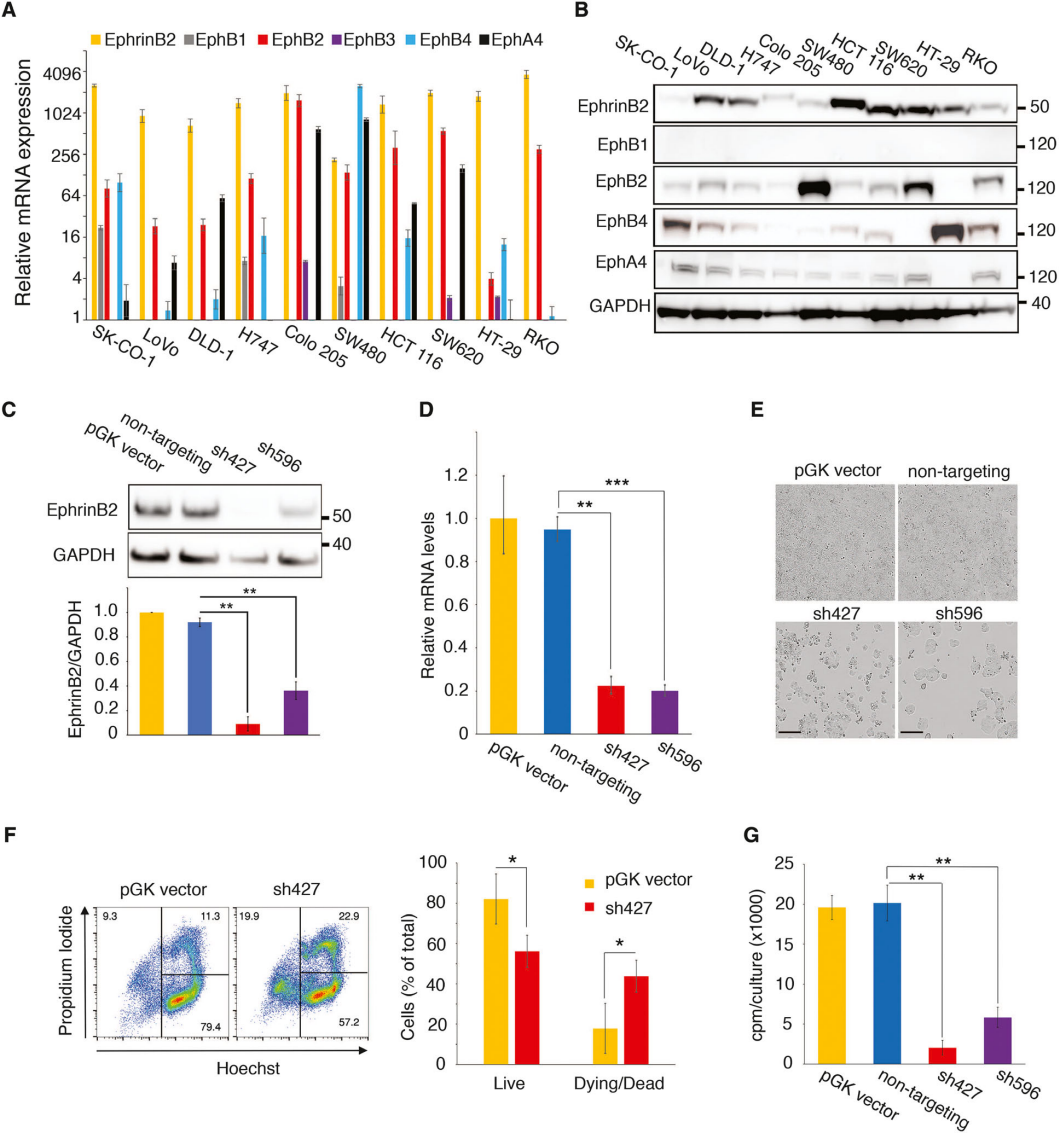
EphrinB2 silencing induces colorectal carcinoma cell death. Relative mRNA (A) and protein (B) levels of EphrinB2 and Eph receptors in the indicated cell lines. The results reflect the mean (±SD shown as error bars) of triplicate determinations by qPCR; proteins are revealed by immunoblotting. (C–E) EphrinB2 was silenced in HT‐29 cells with sh427 and sh596; empty vector (pGK) and non‐targeting shRNA are controls. Representative results (72‐h post‐transduction) from immunoblotting (top) and band quantitation of three independent experiments (bottom) (C); relative mRNA expression (±SD; triplicate determinations) (D); and bright‐field imaging; 20× magnification; scale bars 200 μm (E). (F) HT‐29 cell death following EphrinB2 silencing with sh427; left: representative flow cytometry profiles showing the % viable cells (lower right quadrant) and the remainder non‐viable cells (left panel); right: quantitative results (mean % ± SD, 3 experiments). (G) Reduced proliferation of HT‐29 cells after silencing with sh427 and sh596 compared to controls (pGK vector and non‐targeting shRNA). Representative results expressed as mean counts per minute (cpm) ± SD (triplicate cultures). Statistical significance of group differences determined by two‐tailed Student's *t*‐test; *P < 0.05; **P ≤ 0.01; ***P ≤ 0.001.

To gain mechanistic insight into potential roles of EphrinB2 and its receptors in colorectal carcinoma, we evaluated a panel of 10 well‐characterized human colorectal carcinoma cell lines. Six of these lines (SK‐CO‐1, LoVo, DLD‐1, H747, Colo205, and SW‐620) are of metastatic derivation, four (LoVo, DLD1, HCT 116, and RKO) display microsatellite instability (Berg *et al*., 2017), three are classified as consensus molecular subtype (CMS)‐1 (LoVo, HCT 116, and RKO), and the remainder are either not CMS classifiable (SW620, HT‐29, SK‐CO‐1, Colo 205) or not classified (Linnekamp *et al*., 2018). We found that all cell lines express EphrinB2 mRNA (Fig. 2A) and protein (Fig. 2B). The receptors EphB2, EphB4, and EphA4 mRNAs and proteins were variously detected among the 10 cell lines (Fig. 2A,B). EphB1 and EphB3 mRNAs were generally low in all cell lines, as noted previously (Jagle *et al*., 2014). We did not detect EphB1 protein (EphB1 protein was recognized in controls, Fig. S1A) and our antibodies did not allow consistent detection of human EphB3.

We silenced EphrinB2 expression by using EphrinB2 shRNAs. Evaluation of four different EphrinB2 shRNAs indicated that two of these (sh427 and sh596) reduced EphrinB2 protein levels in HT‐29 cells more efficiently than the other two (sh426 and sh588; Fig. S1B), which led us to use sh427 and sh596 in subsequent experiments. The lower levels of EphrinB2 protein (Fig. 2C) and mRNA (Fig. 2D) in HT‐29 infected with sh427 and sh596 was associated with a sparse cell monolayer that contrasted with the confluent monolayer of control (empty vector or nontargeting shRNA) cells (Fig. 2E). Flow cytometry documented a reproducible increase in cell death (Fig. 2F) and reduction in cell proliferation (Fig. 2G) after EphrinB2 silencing. These results show that sustained EphrinB2 expression is required to maintain the viability of HT‐29 cells.

### 3.3 Effects of EphB2 and EphB4 silencing on colorectal carcinoma cell growth and survival

EphrinB2 functions are classically mediated through EphB receptor ‘forward’ signaling (Kania and Klein, 2016).

**Figure 3:**
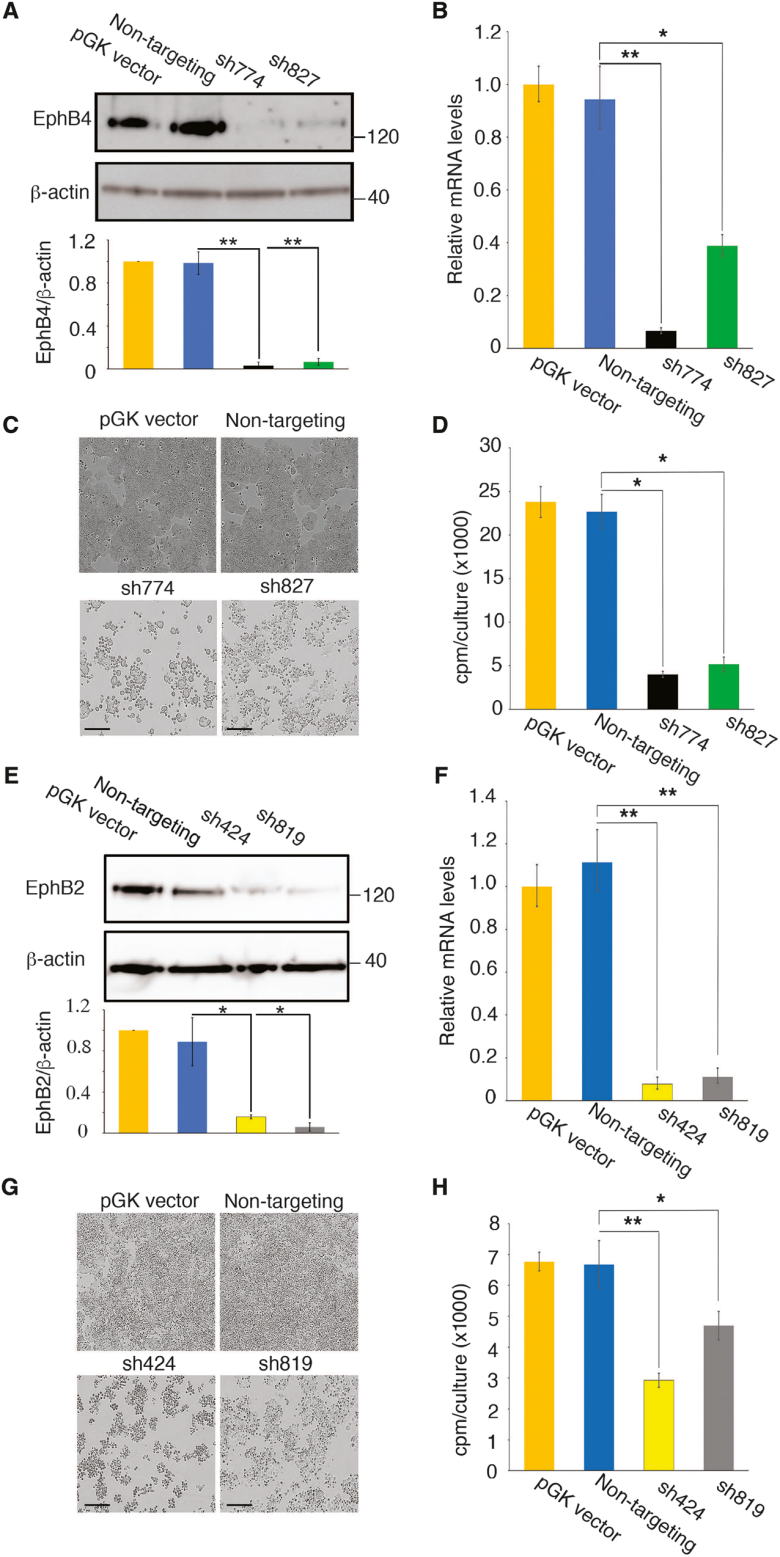
Effects of EphB4 and EphB2 silencing on colorectal carcinoma cell growth and survival. (A–D) EphB4 was silenced in HT‐29 cells with sh774 and sh827; controls included the empty pGK vector and a non‐targeting shRNA. Results 72 h post‐transduction. (A) Representative immunoblotting results (top) and relative band quantitation of three independent experiments (bottom); (B) relative mRNA levels by qPCR (±SD of triplicate determinations); (C) bright‐field images; scale bars 200 μm; and (D) proliferation expressed as mean counts per minute (cpm) ± SD (triplicate cultures). (E–H) EphB2 was silenced 72 h prior to testing in SW620 cells with sh424 and sh819. (E) Representative results from immunoblotting (top) and relative band quantitation of 3 independent experiments (bottom); (F) relative mRNA levels by qPCR (±SD of triplicate determinations); (G) bright‐field images; scale bars 200 μm; and (H) proliferation expressed as mean cpm ±SD (triplicate cultures). Statistical significance of group differences determined by two‐tailed Student's *t*‐test; *P < 0.05; **P ≤ 0.01.

The HT‐29 colorectal carcinoma cell line, which needs EphrinB2 to survive (Fig. 2E–G), contains abundant EphB4 but not EphB1, EphB2, EphB3, or EphA4 proteins (Fig. 2A,B), as observed previously (Jagle *et al*., 2014). We silenced EphB4 by infecting HT‐29 cells with sh774 or sh827 lentiviruses, which reproducibly reduced EphB4 protein (Fig. 3A; Fig. S2A) and mRNA (Fig. 3B). This EphB4 silencing resulted in reduced HT‐29 monolayer coverage (Fig. 3C), reduced cell proliferation (Fig. 3D), and increased cell death whose magnitude was comparable to that induced by the silencing of EphrinB2 (Fig. S2B). As most HT‐29 cells display cell surface EphrinB2 and EphB4 (Fig. S2C), the similarity of outcome in cell death that resulted from the silencing of EphrinB2 or EphB4 suggested that EphrinB2/EphB4 interaction is likely critical to sustain HT‐29 cell viability.

To evaluate the effects of EphB2 depletion, we selected the SW620 colorectal carcinoma cell line, which contains abundant EphB2, whereas EphB1, EphB3, and EphB4 are undetectable and EphA4 is detected at low levels (Fig. 2A,B). Noteworthy, the binding affinity of EphA4 for EphrinB2 (203 nm) is an order of magnitude lower than the affinity of EphB2 for EphrinB2 (22 nm) (Qin *et al*., 2010), implying a greater role for EphB2 than EphA4 in EphrinB2‐induced signaling in SW620. EphB2 silencing with sh424, sh819, sh426, or sh423 lentivirus reproducibly reduced EphB2 protein (Fig. 3E, Fig. S2D) and mRNA (Fig. 3F) levels in SW620 cells, associated with reduced SW620 monolayer coverage (Fig. 3G) and reduced cell proliferation (Fig. 3H). Thus, the silencing EphrinB2 in HT‐29 and SW620 cell lines reduces cell proliferation comparably to the silencing of the receptors EphB2 (SW620) or EphB4 (HT‐29), prompting a broader correlative analysis between the silencing of EphrinB2 and its receptors.

### 3.4 EphrinB2 and EphB prosurvival functions in colorectal carcinoma

We silenced the expression of EphrinB2, EphB2, or EphB4 in the panel of 10 colorectal carcinoma cell lines characterized in Fig. 2A,B (Fig. S3). We did not silence EphB1 or EphB3 because the protein was either not detected (EphB1) or not reliably detected (EphB3) in the cell lines. We also did not silence EphA4 because the EphA4^+^ cell lines coexpressed an alternative EphrinB2 receptor with a much greater affinity for EphrinB2 (Noberini *et al*., 2012a; Qin *et al*., 2010).

**Figure 4:**
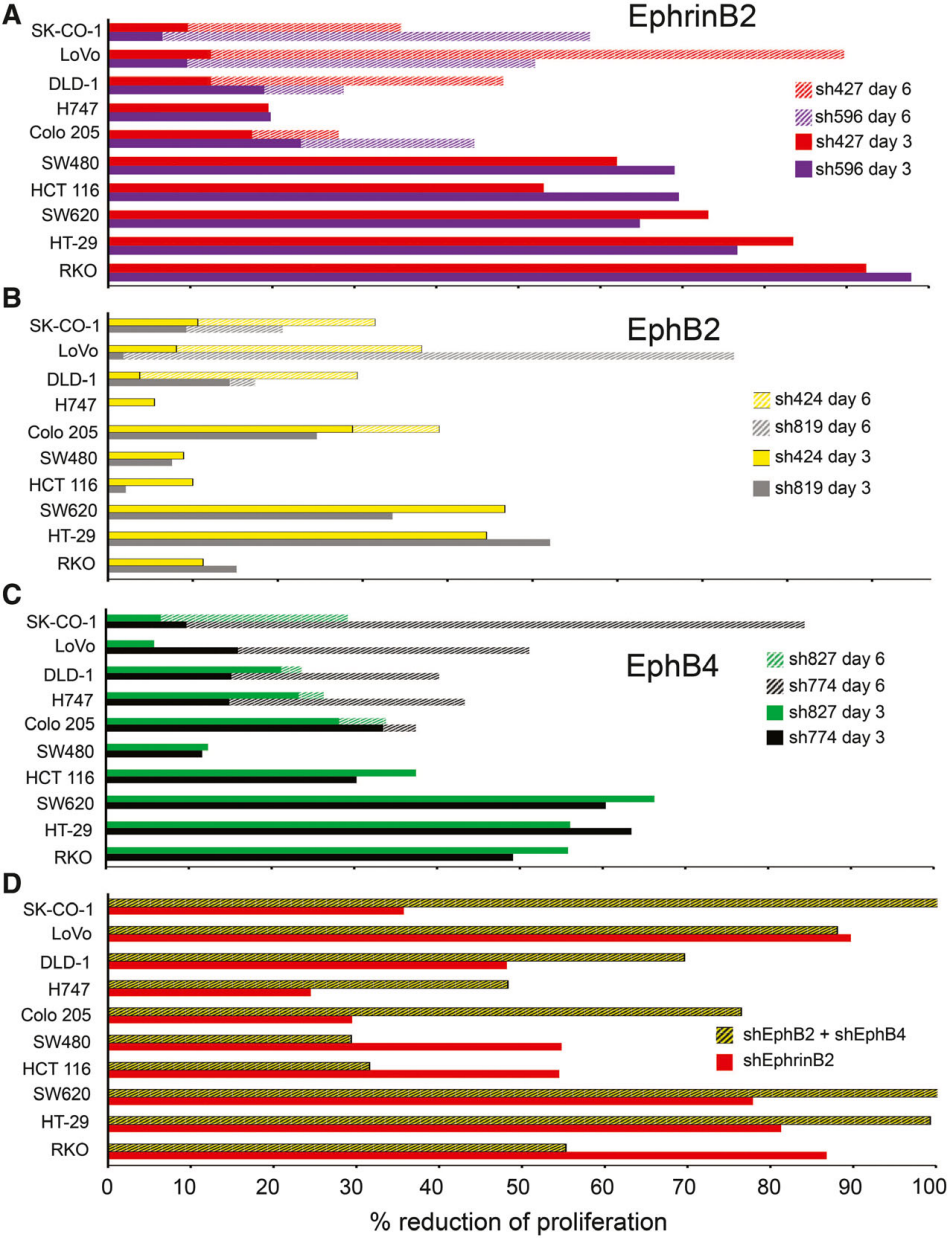
EphrinB2, EphB2, or EphB4 silencing reduces the proliferation of colorectal carcinoma cell lines. (A–C) Relative reduction of cell proliferation 72 (solid) or 144 (dashed) hours after EphrinB2 silencing with sh427 and sh596 (A); EphB2 silencing with sh424 and sh819 (B); or EphB4 silencing with sh774 and sh827 (C). Representative results from triplicate wells. (D) Relative reduction of cell proliferation induced by the silencing of EphrinB2 (sh427) or by the combined silencing of EphB2 (sh424) and EphB4 (sh774). Results of proliferation (72 or 144 h after shRNA infection) expressed as % mean of control (empty vector).

EphrinB2 silencing (sh427 and sh596) reduced by > 50% the proliferation of 7/10 cell lines 3–6 days postinfection and to lower degree the proliferation of the other cell lines (Fig. 4A). The individual silencing of EphB2 (Fig. 4B) and EphB4 (Fig. 4C) reduced substantially (> 50%) the proliferation of 2/10 and 5/10 the cell lines, respectively, and more modestly the proliferation of the remaining cell lines. EphrinB2 silencing was usually more effective at reducing cell proliferation than the silencing of EphB2 or EphB4 (Fig. 4A–C). However, the combined effect from EphB2 plus EphB4 silencing in 7/10 cell lines surpassed or was similar to the inhibition induced by EphrinB2 silencing (Fig. 4D), a result that is consistent with the function of multiple Eph receptors as alternative EphrinB2 receptors.

Thus, EphrinB2 and its receptors EphB2 and EphB4 are general regulators of colorectal carcinoma cell proliferation/viability.

### 3.5 EphB tyrosine kinase inhibition reduces colorectal carcinoma cell growth

The outcome of reduced proliferation/cell death stemming from the silencing of EphrinB2 or its Eph receptors is consistent with the idea that these molecules participate in signaling that sustains cell viability of colorectal cancer cells. Since EphrinB2 and EphB are capable of bidirectional signaling (Kania and Klein, 2016), we sought to discriminate between the effects mediated by receptor and ligand signaling. To this end, we first took advantage of the fact that EphrinB2 does not possess an intrinsic catalytic activity for signaling, relying instead on the recruitment of other molecules to transmit phosphorylation‐dependent and independent signals, whereas the Eph receptors do (Daar, 2012; Kania and Klein, 2016).

**Figure 5:**
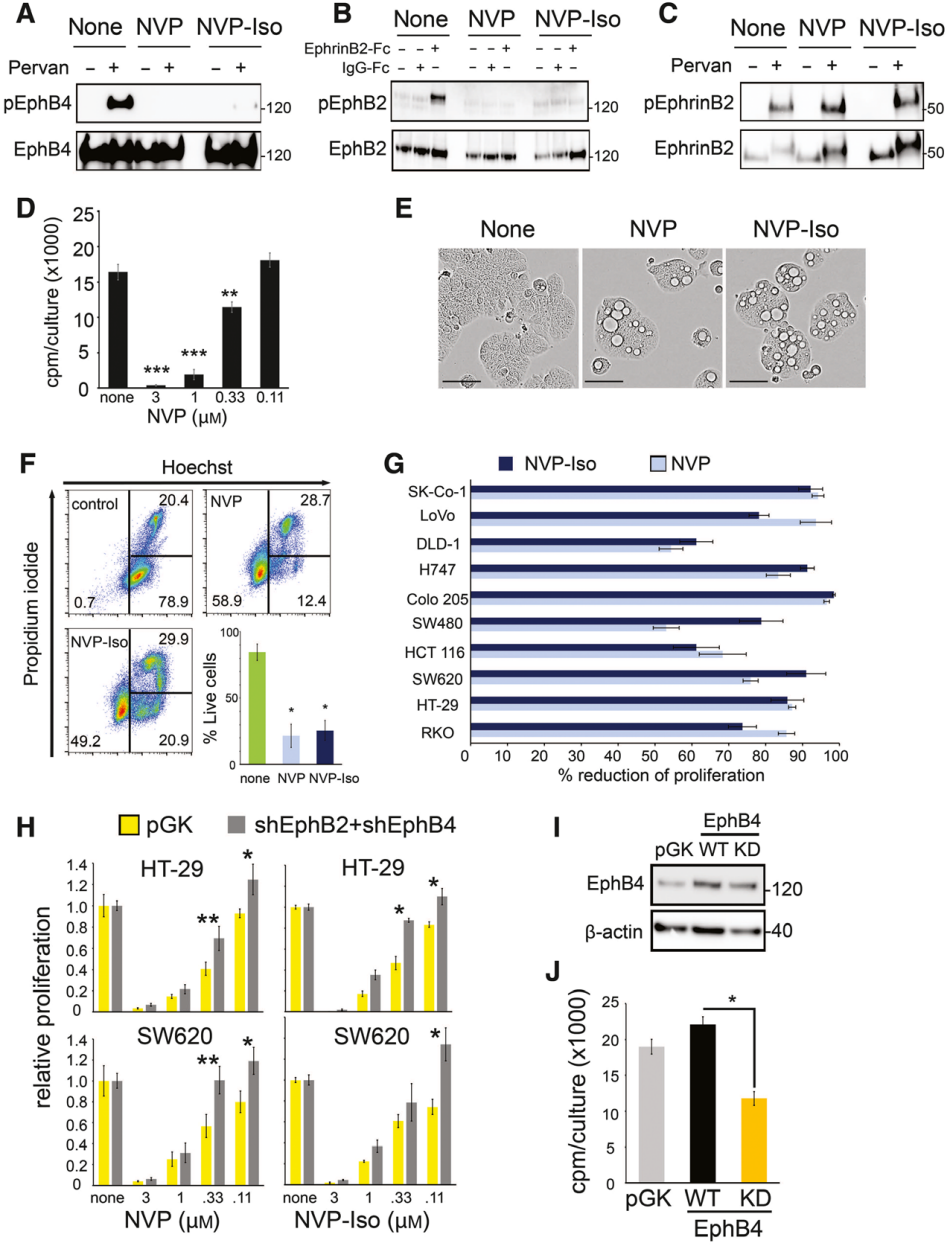
The TKI NVP reduces growth of colorectal carcinoma cells. (A) NVP and NVP‐Iso reduce endogenous EphB4 phosphorylation after 8‐h incubation. EphB4 was immunoprecipitated from HT‐29 cells with or without the tyrosine phosphatase inhibitor pervanadate (Pervan). Immunoprecipitates were immunoblotted with p‐Tyr antibody and reblotted with EphB4 antibody. (B) NVP and NVP‐Iso (4‐h pretreatment) reduce EphB2 phosphorylation induced by EphrinB2‐Fc or control IgG‐Fc in Colo 205 cells. Cell lysates were immunoblotted with p‐EphB2 antibody and reblotted with total EphB2 antibody. (C) NVP and NVP‐Iso (18‐h pretreatment) do not reduce endogenous EphrinB2 phosphorylation. EphrinB2 was immunoprecipitated from HT‐29 cells. Immunoprecipitates were immunoblotted with p‐Tyr antibody and reblotted with total EphrinB2 antibody. (D) NVP (72‐h incubation) dose‐dependently inhibits HT‐29 cell proliferation expressed as mean cpm/triplicate cultures. (E, F) NVP and NVP‐Iso (72‐h incubation) induce morphologic change and cell death in HT‐29 cells. Representative bright‐field images after 3‐day culture; scale bars 50 μm (E). Representative flow cytometry profiles (F); quantitative results (mean % ± SD, 3 experiments). (G) NVP and NVP‐Iso (72‐h incubation) reduce the proliferation of 10/10 colon carcinoma cell lines. Results (3–5 experiments) expressed as % mean (±SEM) of control. (H) Effects of NVP and NVP‐Iso (72‐h incubation) on the proliferation of control (pGK vector, yellow bar) or EphB2 (sh424) plus EphB4 (sh774) silenced (gray bars) HT29 or SW620 cells (silencing 24‐h prior to addition of drugs); results expressed as proliferation relative to no drug. (I, J) Effects of EphB4 WT or KD mutant on the spontaneous proliferation of HT29 cells. Immunoblotting (72 h after transduction) with antibody to EphB4 (I). Proliferation is expressed as mean cpm/culture (J). Statistical significance of difference calculated by two‐tailed Student *t*‐test. *P < 0.05; **P ≤ 0.01; ***P ≤ 0.001.

NVP‐BHG712 is a small molecular weight tyrosine kinase inhibitor (TKI) identified by modeling the EphB4 kinase domain and optimization for inhibition of EphB4 phosphorylation in cells, which showed great selectivity and potency for EphB receptors among a large panel of receptor tyrosine kinase receptors (Chen *et al*., 2017; Martiny‐Baron *et al*., 2010). In validation experiments, we found that 1 μm NVP‐BHG712 (NVP) and its regioisomer NVP‐Iso (Troster *et al*., 2018) reduce endogenous EphB4 tyrosine phosphorylation in the EphB4^+^ HT‐29 cells (Fig. 5A) and specifically reduce EphB2 tyrosine phosphorylation induced by clustered EphrinB2‐Fc in the EphB2^+^ Colo205 cells (Fig. 5B). Consistent with NVP specificity for targeting tyrosine kinase receptors, 1 μm NVP and 1 μm NVP‐Iso did not reduce endogenous EphrinB2 phosphorylation in HT‐29 cells (Fig. 5C). EphrinB2 does not possess an intrinsic catalytic activity for signaling, relying instead on the recruitment of other molecules to transmit phosphorylation‐dependent and independent signals (Daar, 2012).

NVP (Fig. 5D) and NVP‐Iso (Fig. S4A) dose‐dependently reduced HT‐29 cell proliferation. At 1 μm, NVP, and NVP‐Iso reduced HT‐29 cell monolayer coverage (Fig. S4B), caused development of vesicular‐like structures in HT‐29 cells (Fig. 5E) and promoted significant cell death, as assessed by flow cytometry (Fig. 5F). Neither compound altered cell cycle distribution of HT‐29 cells (Fig. S4C). We extended analysis of the effects of NVP and NVP‐Iso on cell proliferation to include the 10 colorectal carcinomas cell lines in which the silencing EphrinB2, EphB2, or EphB4 significantly reduced cell proliferation (Fig. 4). NVP and NVP‐Iso (1 μm) reduced substantially (> 50% reduction) the proliferation of all colorectal carcinoma cell lines (Fig. 5G). The degree of growth reduction induced by NVP and NVP‐Iso across all colorectal carcinoma cell lines in some cases surpassed the degree of inhibition induced by the silencing of EphrinB2 or the silencing of its individual receptors. This is likely attributable to the expression of Eph tyrosine kinase receptors that are not EphrinB2 receptors (Kania and Klein, 2016) in colorectal carcinoma cells (Herath *et al*., 2012) and to the broad Eph targeting of the TKI NVP (Martiny‐Baron *et al*., 2010). In contrast, NVP and NVP‐Iso minimally reduced the proliferation of primary human endothelial cells (human umbilical vein endothelial cells, HUVEC, and human dermal microvascular endothelial cells, hDMEC) and the human osteosarcoma MG63 cell line at the effective dose (1 μm) (Fig. S4D). Noteworthy, HUVEC express EphrinB2 and EphB4, but HUVEC survival in vitro is independent of EphB4 signaling (Salvucci *et al*., 2015).

To assess further the Eph specificity of NVP and NVP‐Iso, we tested the effects of these inhibitors prior to and after the silencing EphB2 and EphB4 receptors. The results show that NVP and NVP‐Iso were significantly less effective at reducing the proliferation of HT‐29 and SW620 colorectal carcinoma cells after EphB2 (sh424) and EphB4 (sh774) were both silenced compared to the control cells (Fig. 5H). We also expressed a kinase‐deficient (KD) EphB4 mutant (K647R/kdEPHB4; contains a single K647R mutation in the kinase domain), which acts as a dominant negative for the endogenously expressed EphB4 while retaining the ability to activate EphrinB2 signaling (Yang *et al*., 2006) (Fig. 5I and Fig. S4E). Compared with wild‐type (WT) EphB4, the mutant EphB4 reduced the spontaneous proliferation of HT‐29 cells (Fig. 5J). Overall, these results support a critical role of Eph tyrosine kinase signaling in controlling the survival and growth of colorectal carcinoma cells.

### 3.6 Autophagy regulation in colorectal carcinoma cells

The vesicle‐like structures visualized in HT‐29 cells (Fig. 5E), and to varying degrees in most NVP and NVP‐Iso‐treated colorectal carcinoma cell lines (Fig. S5), resemble autophagic vesicles called autophagosomes. Similar vesicular structures also developed after EphrinB2 silencing, especially in those colorectal cell lines most growth inhibited by the silencing of EphrinB2 (Fig. S6), but not after the silencing of EphB2 or EphB4. This result is in line with the more modest growth inhibition induced by the silencing of EphB2 or EphB4 compared to EphrinB2 in the cell lines, and the functional redundancy of Eph receptors.

We tested whether NVP and NVP‐Iso induce autophagy, a cellular stress response that principally serves to regulate the turnover of damaged cell organelles and proteins that become engulfed into autophagosomes (Marino *et al*., 2014). Although autophagy generally promotes cell survival, it can lead to cell death in contexts in which the intensity or duration of the stress response is above a certain threshold (Fulda and Kogel, 2015; Marino *et al*., 2014).

**Figure 6:**
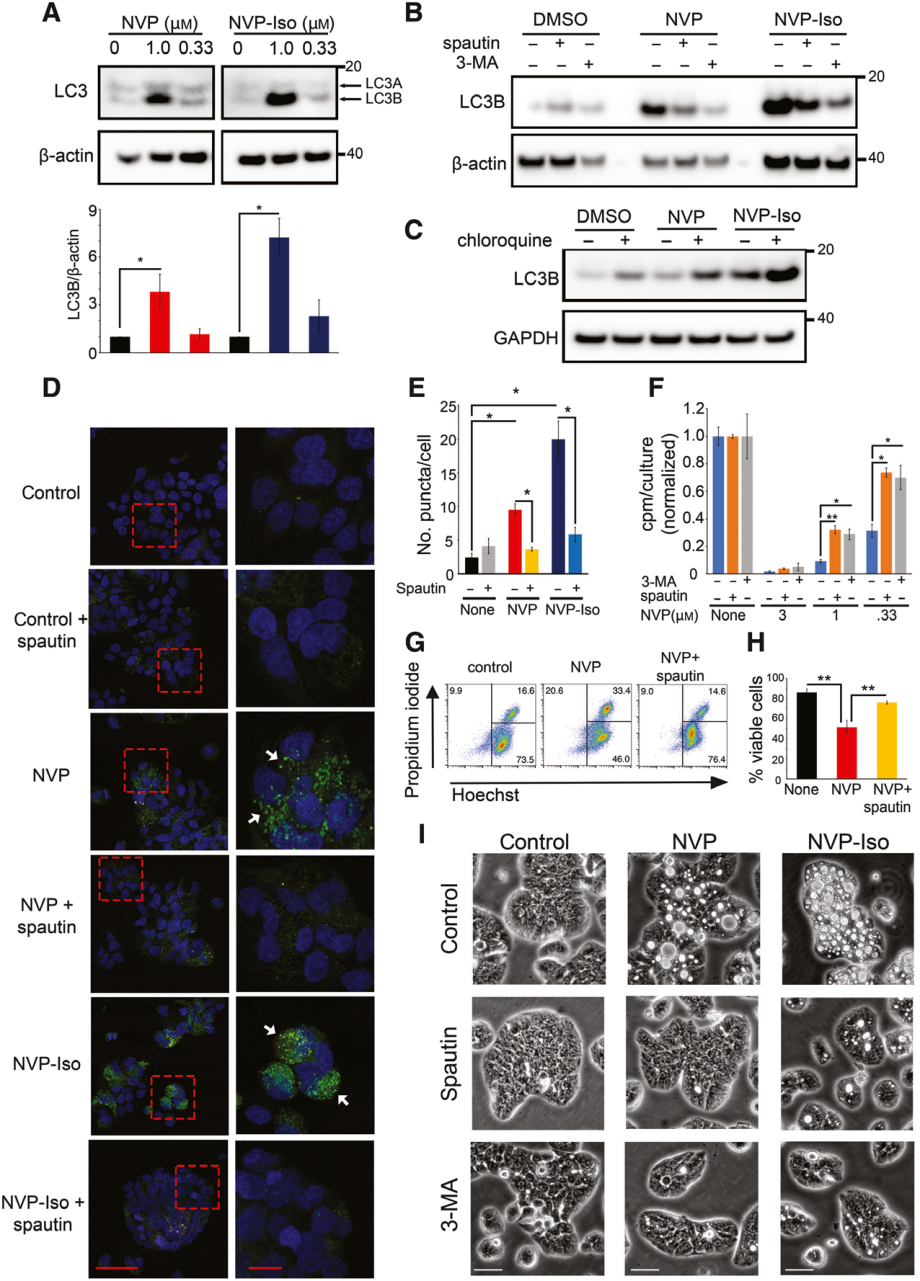
NVP and NVP‐Iso induce autophagy in colorectal carcinoma cells. (A) NVP and NVP‐Iso (72‐h incubation) induce the autophagy marker protein LC3B in HT‐29 cells. LC3 antibody detects LC3A and LC3B (lipidated derivative) in cell lysates of HT‐29 cells. Representative immunoblot (top) and LC3B quantification from three independent experiments (bottom). (B) Spautin and 3‐MA (4‐h preincubation) reduce LC3B levels in HT‐29 cells treated with NVP or NVP‐Iso after 72‐h incubation. (C) Chloroquine (4‐h incubation prior to harvest) enhances LC3B levels in HT29 cells treated with NVP or NVP‐Iso (72‐h incubation). (D, E) Autophagosomes are identified as ‘puncta’ (pointed by white arrows) in HT‐29 cells transduced with a fluorescent‐LC3 vector. Spautin (4‐h pretreatment) reduces the number of autophagosomes in NVP and NVP‐Iso treated (72 h) HT‐29 cultures; representative confocal images (63×); boxed areas (left) are magnified (right); scale bars 50 μm (left); 10 μm (right) (D). Quantitative results (E) reflect the mean number of puncta/cell (400–500 total DAPI^+^ cells/3 fields; cellprofiler). (F) Effect of spautin or 3‐MA on HT‐29 cell proliferation inhibited by NVP (72‐h incubation). Results reflect mean cpm/culture ± SD (triplicate cultures). (G, H) Spautin (4‐h pretreatment) reduces cell death of HT‐29 cells incubated with NVP (0.5 μm; 72 h); representative flow cytometry profiles (G) and quantitative results of three independent experiments (mean % ± SD) (H). (I) Spautin and 3‐MA normalize HT29 cell morphology altered by NVP or NVP‐Iso (72 h); representative bright‐field images; scale bars 25 μm. Statistical significance of difference calculated by two‐tailed Student's *t*‐test. *P < 0.05; **P ≤ 0.01; ***P ≤ 0.001.

The light‐chain 3 (LC3) of microtubule‐associated proteins exists in two forms, LC3A and its lipidated form LC3B that is associated with autophagosomal membranes (Kabeya *et al*., 2000). We found that NVP and NVP‐Iso reproducibly induce accumulation of the autophagosome marker LC3B in HT‐29 cells (Fig. 6A). Spautin (5 μm) and 3‐methyladenine (3‐MA; 5 mm), inhibitors of early steps in the autophagic pathway, reduced accumulation of LC3B in HT‐29 cells and other colorectal carcinoma cells treated with 1 μm NVP or NVP‐Iso (Fig. 6B, Fig. S7A). Instead, the autophagic flux inhibitor, chloroquine (CQ; 10 μm), enhanced the accumulation of LC3B in colorectal carcinoma cells treated with 1 μm NVP or NVP‐Iso (Fig. 6C) but decreased autophagy (Fig. S7B) and mitigated the antiproliferative effects of NVP and NVP‐Iso in these cells (Fig. S7C,D). By imaging HT‐29 cells that were stably transduced with pK‐fluorescent LC3, we visualized the accumulation of LC3 in autophagic ‘puncta’ after treatment with NVP or NVP‐Iso, which was reduced by spautin (Fig. 6D). Image quantitation showed that 1 μm NVP and NVP‐Iso increase significantly the average number of LC3‐related puncta/cell and that spautin reduces this drug effect (Fig. 6E).

We also documented that EphrinB2 silencing induces the accumulation of LC3B in HT‐29 cells (Fig. S7E) and the accumulation of LC3 autophagic ‘puncta’, which were inhibited by spautin (Fig. S7F). EphB4 silencing in HT‐29 cells induced the accumulation of Atg5 (autophagy‐related 5), a protein required for autophagic vesicles formation (Fig. S7G). Thus, NVP, NVP‐Iso, and EphrinB2 silencing induce autophagy in colorectal carcinoma cells.

We examined whether autophagy plays a causal role in the induction of cell death/reduced cell proliferation induced by NVP and NVP‐Iso. If autophagy plays such role, inhibitors of early events in the autophagic pathway, such as spautin and 3‐MA, would be expected to mitigate the antiproliferative and pro‐apoptotic effects of NVP and NVP‐Iso. Both inhibitors mitigated the antiproliferative (Fig. 6F, Fig. S7H) and death‐promoting effects of NVP and NVP‐Iso (Fig. 6G,H; Fig. S7I,J) in colorectal cancer cells. In addition, bright‐field imaging documented that spautin and 3‐MA protect colorectal carcinoma cells from acquiring the typical vesicular appearance induced by treatment with NVP or NVP‐Iso and allows the cells to grow (Fig. 6I).

Additionally, the autophagy inducer rapamycin (rapa) (Fulda and Kogel, 2015; Marino *et al*., 2014) induced accumulation of LC3B in colorectal carcinoma cells, albeit to a somewhat lower degree than NVP and NVP‐Iso, and expectedly reduced constitutive activity of the mammalian target of rapamycin that was not altered by NVP and NVP‐Iso (Fig. S8A). Rapa induced cell death (Fig. S8B) and reduced proliferation of colorectal carcinoma cells (Fig. S8C,D) comparably to NVP and NVP‐Iso, but the typical vesicular morphology induced by NVP and NVP‐Iso was muted in rapa‐treated cells (Fig. S8E). Confirming the results with the autophagy inhibitors spautin and 3‐MA, ATG5‐depleted colorectal carcinoma cells had a reduced content of LC3B after exposure to NVP or NVP‐iso compared to control nonsilenced cells (Fig. S8F). In addition, cell death associated with a vesicular morphology was reduced in ATG5‐depleted colorectal cells that had been treated with NVP compared to control cells (Fig. S8G,H).

Furthermore, the irreversible pan‐caspase inhibitor Z‐VAD‐FMK (ZVAD) did not reconstitute colorectal cell proliferation (Fig. S8I) or viability (Fig. S8J) reduced by NVP or NVP‐Iso. In sum, these experiments provide evidence that autophagy is the cell death pathway induced by Eph tyrosine kinase inhibition or the silencing of EphrinB2 ligand in colorectal cancer cells.

### 3.7 EphB tyrosine kinase inhibition reduces colorectal carcinoma growth in mice

Since a mouse model reproducing key features of human colorectal carcinoma is not currently available (Romano *et al*., 2018), we tested the effects of NVP‐Iso on the growth of human colorectal carcinoma cells in immunodeficient mice (BALB/c nu/nu; female; 6–10 weeks of age). We separately injected subcutaneous (s.c.) Colo205 and HT‐29 colorectal carcinoma cells (10 × 10^6^ cells/mouse). After the tumors reached an average volume of 100 mm^3^ (*V* = 1/2 × *D* × *d* ^2^), the mice were randomized to receive daily intraperitoneal (i.p.) injections of NVP‐Iso (15 mg·kg^−1^; 10 mice) or vehicle only (10 mice). The diet was supplemented with gel meal. The experimental endpoint was time for any tumor to reach a maximum diameter of 20 mm in any direction. A daily regimen was selected on the basis of prior pharmacokinetics studies (Martiny‐Baron *et al*., 2010), and the dose and route of administration were selected on the basis of our preliminary experiments showing that a dose of 30 mg·kg^−1^ i.p. caused significant reduction of body weight and that the oral route of administration previously tested (Martiny‐Baron *et al*., 2010) was poorly tolerated (not shown).

**Figure 7:**
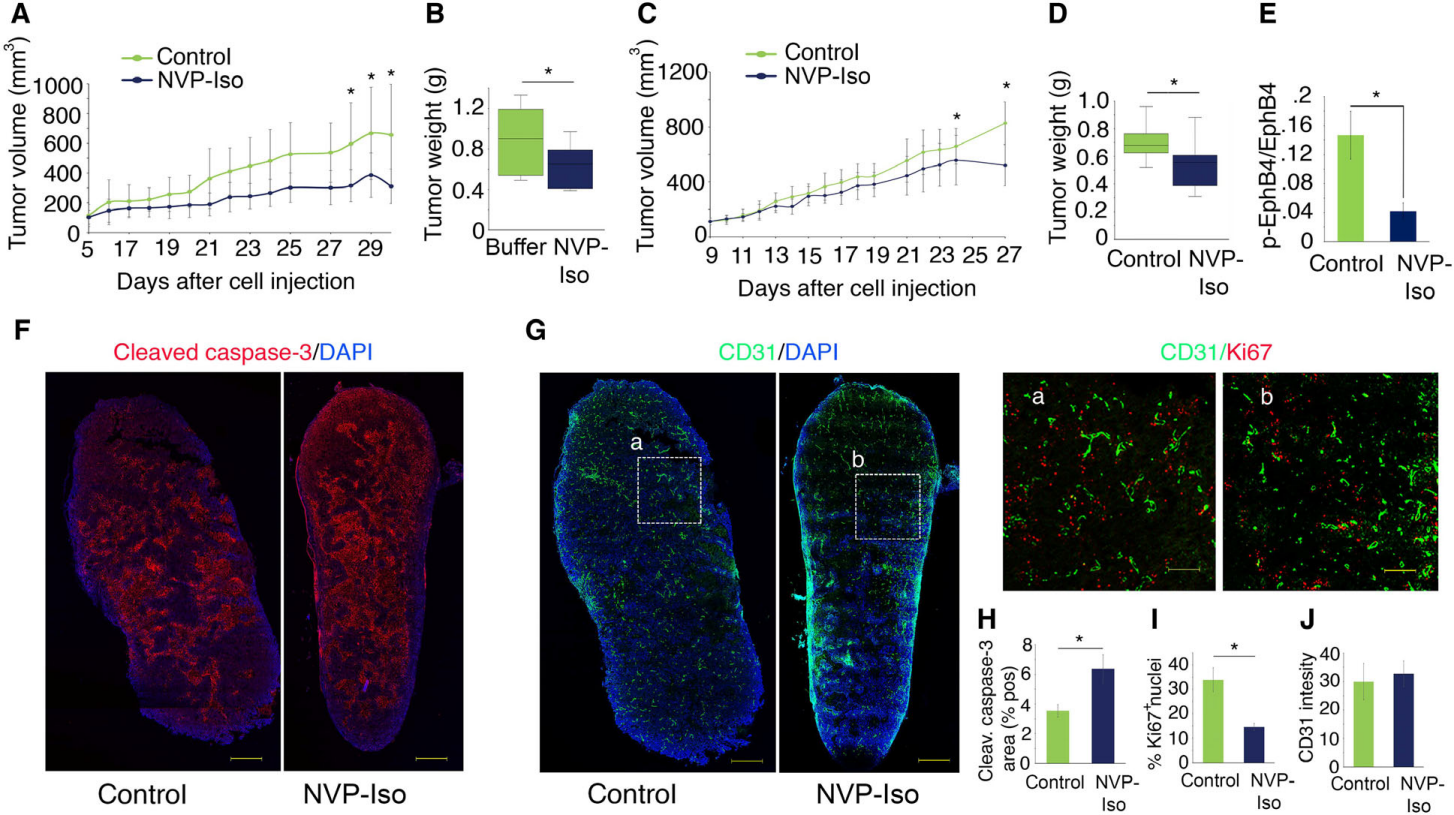
The Eph TKI NVP‐Iso reduces colorectal cancer growth. (A–D) Colo 205 (A, B) or HT‐29 (C, D) cells (10 × 10^6^) were injected s.c. in nu/nu mice. When the average tumor volume reached 100 mm^3^, mice (10/group) were randomized to receive daily i.p. injections of NVP‐Iso (15 mg·kg^−1^) or vehicle only. Results show the average tumor volume (±SD) as a function of time from tumor cell injection (A, C) and tumor weight after tumor harvest (B, D). Tumor weight results are displayed as box‐and‐whisker plots; the horizontal line in the box reflects the median tumor weight. (E) HT‐29 tumor extracts from control or NVP‐Iso‐treated mice (experiment in panel D) were tested for tyrosine‐phosphorylated EphB4 and total EphB4 content. Results are expressed as the mean (±SD) ratio of tyrosine‐phosphorylated EphB4/total EphB4 (measured in pg from 50 μg tumor lysate; 10 drug‐treated mice and 10 controls tested). (F, G) Cleaved caspase‐3 (red) (F); CD31 (green) and Ki67 (red) (G) immunostaining of representative HT29 tumor sections from control and NVP‐Iso‐treated mice; cell nuclei (DAPI^+^) are blue. Tumors were removed after completion of treatment (experiment in panel D). Boxed tumor areas (a and b) are magnified on the right of panel (G). Scale bars 1000 μm (F, G); 200 μm (magnified panels in G). (H–J) Quantitation of cleaved caspase‐3^+^ (H), Ki67^+^ (I), and CD31^+^ (J) immunostaining in control (*n* = 5) and NVP‐Iso‐treated (*n* = 5) tumors (experiment shown in C, D). Results are expressed as: mean % (±SD) cleaved caspase‐3^+^ tumor area; mean % (±SD) Ki67^+^ cell nuclei in tumor sections; and average (±SD) CD31^+^ fluorescence intensity in tumor sections; *P < 0.05.

NVP‐Iso reduced significantly Colo205 tumor growth as determined by tumor measurements (Fig. 7A) and tumor weight (Fig. 7B). NVP‐Iso also reduced significantly the growth of HT‐29 colorectal carcinoma cells in mice (Fig. 7C,D). Although significant, the antitumor effect of NVP‐Iso *in vivo* was lower in magnitude than expected from the results *in vitro* with the tumor cell lines. We examined tyrosine‐phosphorylated Eph in tumor tissue extracts. As shown (Fig. 7E), the relative levels of tyrosine‐phosphorylated EphB4 were significantly lower in HT‐29 tumor extracts from NVP‐Iso‐treated mice compared to controls, but residual tyrosine phosphorylation was detected despite treatment. This suggested insufficient dosing through the i.p. route of administration, which we could not rectify due to drug toxicity at higher concentrations.

Based on the *in vitro* results showing that NVP and NVP‐Iso promote cell death and reduces cell proliferation, we examined these parameters in tumors removed from the mice at the end of treatment. Representative HT‐29 sections encompassing entire tumors through their maximum diameter show that cleaved caspase‐3 (cell death marker) is more widely detected in the NVP‐Iso‐treated tumor compared to the control (representative tumor, Fig. 7F), and that the cell replication marker Ki67 is more widely detected in the control compared to the NVP‐Iso‐treated tumor (same representative tumor, Fig. 7G, magnified panels a and b). Confirming these observations, quantitative results show that the mean % cleaved caspase‐3^+^ area is significantly greater in NVP‐Iso‐treated mice (*n* = 5) compared to control (*n* = 5) (Fig. 7H) and that the average % Ki67^+^ cells in the control (*n* = 5) is significantly higher compared to the NVP‐Iso‐treated (*n* = 5) tumors (Fig. 7I). Within viable portions of the HT‐29 tumors, the distribution and morphology of the vasculature, based on immunostaining of the endothelial CD31 marker, appear similar in the control and drug‐treated tumors (Fig. 7G). This interpretation was confirmed by measurement of the average CD31‐derived fluorescence intensity in control (*n* = 5) and NVP‐Iso‐treated tumors (*n* = 5) (Fig. 7J), indicating that NVP‐Iso has no measurable effect on the tumor vasculature. Consistent with these findings, CD31^+^ vascular structures with normal morphology were visualized even within cleaved caspase 3^+^ tumor areas of drug‐treated mice, and NVP‐Iso did not reduce the proliferation of primary endothelial cells in culture (Fig. S4B), suggesting that tumor tissue degeneration is not attributable to a primarily vascular effect of the drug. Overall, these results show that inhibition of the Eph tyrosine kinase inhibits the growth of human colorectal carcinomas.

## 4 Discussion

This study provides novel insights into colorectal carcinoma cell survival and delineates a novel therapeutic strategy. We made three observations. First, we discovered that phosphotyrosine‐dependent signaling from EphB receptors sustains colorectal carcinoma cell survival. Consistent with the current results, previous observations noted that EphA2 signaling protects breast cancer cells from death (Harada *et al*., 2011), EphB3 signaling suppresses Fas‐induced apoptosis in T cells (Maddigan *et al*., 2011), and the kinase inhibitor AZ12672857 reduces the viability of selected colorectal carcinoma cells (McCall *et al*., 2016). Thus, the current observation extends the spectrum of EphB receptors functions. This newly appreciated function of EphB is particularly relevant to colorectal carcinoma where EphrinB2 and its Eph receptors are widely expressed providing many opportunities for Ephrin/Eph interaction and Eph activation.

The second observation we made is that autophagy is the process leading to colorectal cancer cell death when Eph receptor tyrosine kinase (RTK) signaling is inhibited. This unveils a previously unrecognized role of Eph RTKs as inhibitors of autophagy in colorectal carcinoma cells. RTK signaling and autophagy are recognized as interconnected processes, but the interplay between these pathways appear complex and context‐dependent (Levy *et al*., 2017). In non‐small‐cell lung cancer (NSCLC) with oncogenic mutations of epidermal growth factor receptor, autophagy contributed to the efficacy of TK inhibitors (TKI) by reducing tumor cell viability (Wei *et al*., 2013), but in RAS‐driven NSCLC, depletion of the autophagy protein Atg7 blocked tumor growth (Karsli‐Uzunbas *et al*., 2014).

The third observation we made is that the Eph TKI NVP‐Iso inhibited significantly and reproducibly the growth of human colorectal carcinoma in mice attributable to a drug effect on the tumor cells. NVP was reported to inhibit VEGF‐induced angiogenesis (Martiny‐Baron *et al*., 2010), but we did not detect an effect of NVP or NVP‐Iso on endothelial cell growth or the tumor vasculature. The antitumor effect of NVP‐Iso was lower than what we predicted from its potency *in vitro*. Several reasons could account for this, including intrinsic differences between experiments *in vitro* that fail to capture the complexities of a protumorigenic microenvironment, suboptimal dose/regimen, or emergence of resistance to treatment.

Colorectal cancer is a leading cause of death worldwide. Despite therapeutic improvements, advanced colorectal cancer is not currently curable (Welch and Robertson, 2016). Regorafenib, a TKI that predominantly targets angiogenesis‐related signaling, is the only TKI approved for the treatment metastatic colorectal cancer (Matos *et al*., 2016). Here, we show that Eph signaling sustains colorectal carcinoma cell survival and growth and that inhibition of the phosphotyrosine‐dependent Eph signaling is effective at blocking this prosurvival function. Existing Eph kinase inhibitors and others currently under development (Boyd *et al*., 2014; Chen *et al*., 2017) may provide new therapeutic opportunities for colorectal carcinoma.

## 5 Conclusions

These results show that Eph receptor tyrosine kinase‐dependent signaling is a previously unrecognized pathway that sustains colorectal carcinoma cell survival preventing autophagy‐mediated cell death and represents a therapeutic target in colorectal carcinoma.

## Conflict of interest

The authors declare no conflict of interest.

## Author contributions

Conception and design: GT and MD. Development of methodologies: MD, GT, DM, NT‐G, DS‐M, and HK. Acquisition of data: MD, DW, and NT‐G. Analysis and Interpretation of data: MD, DW, and GT. Administrative, technical, or material support: AT, DK, HS.

## Supporting information


**Fig. S1.** EphB1 protein detection and EphrinB2 silencing by shRNA.
**Fig. S2.** EphB4 and EphB2 detection and silencing in colorectal carcinoma cell lines.
**Fig. S3.** EphrinB2, EphB2, EphB4 silencing in colorectal carcinoma cell lines.
**Fig. S4.** NVP‐Iso cell proliferation, cell cycle analysis, negative control cell lines and EphB4 overexpression mRNA levels.
**Fig. S5.** Morphologic changes in colorectal carcinoma cell lines with NVP or NVP‐Iso.
**Fig. S6.** Morphologic changes in colorectal carcinoma cell lines after EphrinB2 silencing.
**Fig. S7.** Autophagy markers in colorectal carcinoma cells.
**Fig. S8.** Autophagy in colorectal carcinoma cells.
**Table S1.** Primers for qRT‐PCR.
**Table S2.** Lentiviral constructs.
**Table S3.** List of antibodies used.
