# Supplementary material for: Identification of Eph receptor signaling as a regulator of autophagy and a therapeutic target in colorectal carcinoma
Source: Mol Oncol. 2019 Oct 23;13(11):2441–59. doi: 10.1002/1878-0261.12576 (PMC6822245; doi:10.1002/1878-0261.12576)
Supplement: Supplementary file 1 — Fig. S1. EphB1 protein detection and EphrinB2 silencing by shRNA. Fig. S2. EphB4 and EphB2 detection and silencing in colorectal carcinoma cell lines. Fig. S3. EphrinB2, EphB2, EphB4 silencing in colorectal carcinoma cell lines. Fig. S4. NVP‐Iso cell proliferation, cell cycle analysis, negative control cell lines and EphB4 overexpression mRNA levels. Fig. S5. Morphologic changes in colorectal carcinoma cell lines with NVP or NVP‐Iso. Fig. S6. Morphologic changes in colorectal carcinoma cell lines after EphrinB2 silencing. Fig. S7. Autophagy markers in colorectal carcinoma cells. Fig. S8. Autophagy in colorectal carcinoma cells. Table S1. Primers for qRT‐PCR. Table S2. Lentiviral constructs. Table S3. List of antibodies used. [file MOL2-13-2441-s001.pdf]

Supporting information for:

Identification of Eph receptor signaling as a regulator of autophagy and a therapeutic target in colorectal carcinoma

Michael DiPrima, Dunrui Wang, Alix Tröster, Dragan Maric, Nekane Terrades-Garcia, Taekyu Ha, Hyeongil Kwak, David Sanchez-Martin, Denis Kudlinzki, Harald Schwalbe and Giovanna Tosato

The file Includes:

Supporting tables 1-3

Supporting figures 1-8

Table S1. Primers used for quantitative PCR

Target	Forward	Reverse
GAPDH	CATgAgAAgTATgACAACAgCCT	AgTCCTTCCACgATACCAAAgT
EFNB1	AAgAACCTggAgCCCgTATC	gCAgATgATgTCCAgCTTgT
EFNB2	TggggTgTTTTgATggTTTT	CCAgTCCTTgTCCAggTAgAA
EFNB3	CAgCCTggAgCCTgTCTACT	CgATCTgAgggTACAgCACA
EphB1	gCTACCTCCAggATgACACC	gACgATCCCATAgCTCCAAA
EphB2	TgTgTAACAgACgggggTTT	CCTCgTAggTgAAAggATCg
EphB3	TgCCACTCAAgCTCTACTgC	CTgCTTCgCCTTgTAgCTC
EphB4	CTTTggAAgAgACCCTgCTg	TgCACgTCACACACTTCgTA
EphB6	ggCTgTTAATggggTgTCTg	gACAgCAgAgggCACTTCAT
EphA4	TCATTggAgATggggCTAAC	CCgCTTCTTgTTTggCTTTA

Table S2. Lentiviral Constructs

Target	Name	TRC[A] number Cat. no.	Target region[B]	Target sequence
EphB4	#774	TRCN0000001774	CDS	TGATCTGAAGTGGGTGACATT
EphB4	#651	TRCN0000010651	CDS	CTGGAGTTACGGGATTGTGAT
EphB4	#826	TRCN0000314826	CDS	ACAGCATGCCCGTCATGATTC
EphB4	#407	TRCN0000195407	CDS	CGTCATGATTCTCACAGAGTT
EphB4	#827	TRCN0000314827	3UTR	CACCACCAAACTCAATCATTT
EphB2	#424	TRCN0000006424	CDS	GCTGTGATTTCCAGTGTCAAT
EphB2	#819	TRCN0000199819	CDS	GCGTGTCTTCTACCGCAAGTG
EphB2	#426	TRCN0000006426	CDS	CGGGAGTTTGCCAAGGAAATT
EphB2	#423	TRCN0000006423	CDS	GCTAGACAAGATGATCCGCAA
EphB2	#573	TRCN0000196573	CDS	GAAGATCTACATCGATCCTTT
EphrinB2	#427	TRCN0000058427	CDS	CTGGTACTATACCCACAGATA
EphrinB2	#423	TRCN0000058423	CDS	CCAGGAATAAAGATCCAACAA
EphrinB2	#426	TRCN0000058426	CDS	CCTTATTTGCAGGGATTGCTT
EphrinB2	#596	TRCN0000285596	3UTR	CTTTCCCAGAGGACACCTAAT
EphrinB2	#588	TRCN0000276588	CDS	TCTACATCAAATGGGTCTTTG
plKO	Control	SHC001		no insert
non-targeting	Non-targeting	SHC002		CAACAAGATGAAGAGCACCAA
pk-hLC3B	pk-LC3	61460 (Addgene)		

All constructs purchased from Sigma, unless otherwise noted

A, The RNAi Consortium; B, Coding region or 3'UnTranslated Region

Table S3. List of antibodies used

Target	Host	Clonality^{A}	Source	Catalog number	Western dilution	IF^{B} dilution	IP^{C} per 250μg lysate
EphrinB2	rabbit	mono	Abcam	ab150411	1:1000	1:100	
EphrinB	rabbit	poly	Santa Cruz	sc-910			1μg
pEphrinB	rabbit	poly	Cell Signaling	3481		1:100	
EphA4	rabbit	poly	Abcam	ab5396	1:1000		
EphB1	rabbit	poly	Stratagene		1:500		
EphB2	goat	poly	R&D Systems	AF467	1μg/mL		
pEphB1+B2 (pY594+pY604)	rabbit	poly	Abcam	ab61791	1:500		
EphB4	goat	poly	R&D Systems	AF3038	1μg/mL	5μg/mL	1μg
GAPDH	mouse	mono	Santa Cruz	sc-47724	1:1000		
B-actin	mouse	mono	Santa Cruz	sc-47778	1:1000		
pTyr (4G10)	mouse	mono	Millipore-Sigma	05-1050	1:1000		
CD31	rat	mono	HistoBiotec	SZ31		1:20	
KI67	rabbit	mono	Cell Signaling	9129		1:400	
cleaved Caspase-3	rabbit	mono	Cell Signaling	9579		1:200	
LC3 A/B	rabbit	mono	Cell Signaling	12741	1:1000		
ATG5	rabbit	mono	Cell Signaling	12994	1:1000		
p-mTor (S2448)	rabbit	poly	Cell Signaling	2971	1:1000		
mTor	mouse	mono	Cell Signaling	4517	1:1000		

A, mono, monoclonal; poly, polyclonal; B, immunofluorescence; C IP, immunoprecipitation

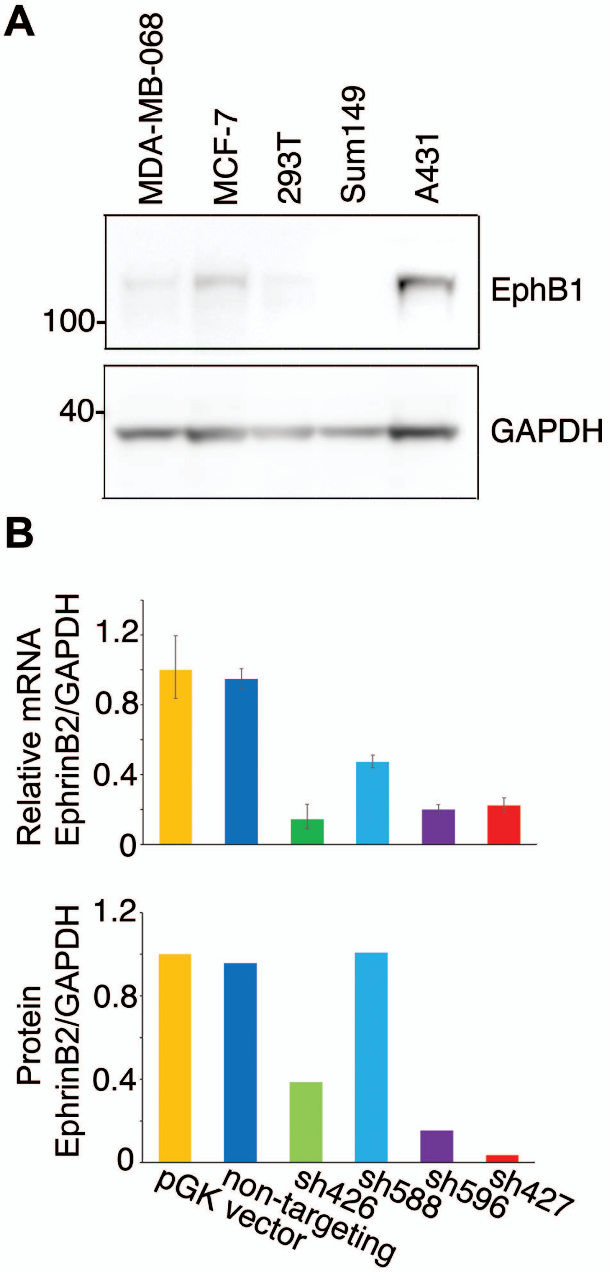

Fig. S1. EphB1 protein detection and EphrinB2 silencing by shRNA. (A) Specific EphB1 detection by immunoblotting in the indicated cell lines; GAPDH is used as a control. (B) Effects of EphrinB2 shRNAs and controls (empty vector and non-targeting shRNA) on relative levels of EphrinB2 mRNA expression by qPCR (top bar graph) and relative EphrinB2 protein levels detected by immunoblotting and band quantitation in HT-29 cells (bottom bar graph). Results at 72 hours after lentiviral infection.

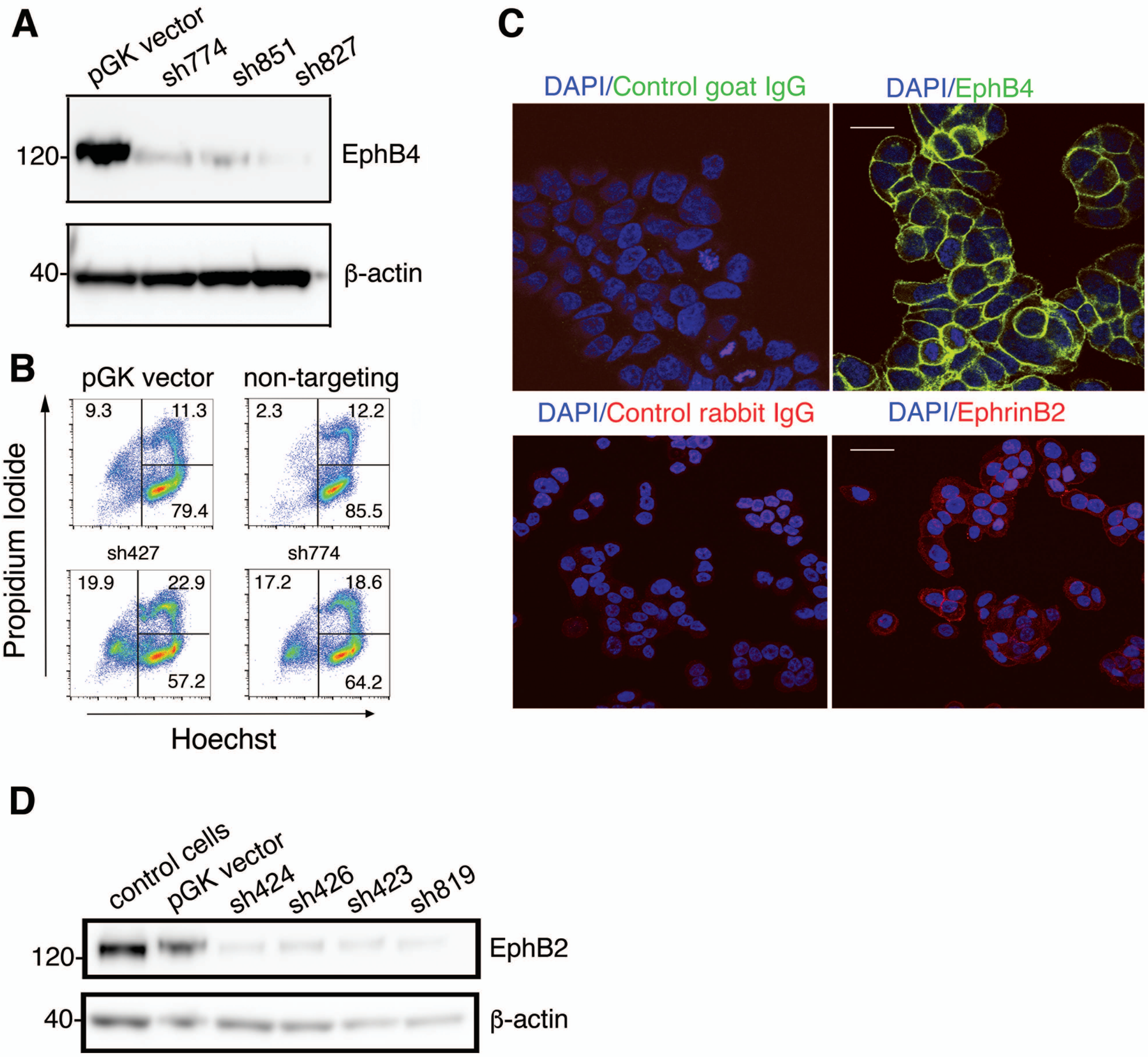

Fig. S2. EphB4 and EphB2 detection and silencing in colorectal carcinoma cells. (A) EphB4 protein levels are specifically reduced in HT-29 cells by sh774, sh851 and sh827; immunoblotting results; β-actin reblotting used as a control. (B) Reduced HT-29 cell viability 72 hours after EphB4 silencing with sh774 and EphrinB2 silencing with sh427. Representative flow cytometry profiles showing reduced HT-29 cell viability (percent live cells in the lower right quadrant) after EphB4 silencing. (C) Specific immunohistochemical detection of EphB4 (green) and EphrinB2 (red) in HT-29 cells. Nuclei are stained with DAPI (blue). Representative confocal images; scale bar 20μm (top), 33μm (bottom). (D) EphB2 protein levels are specifically reduced in SW620 cells 72 hours after infection with sh424, sh426, sh423 and sh819; immunoblotting results.

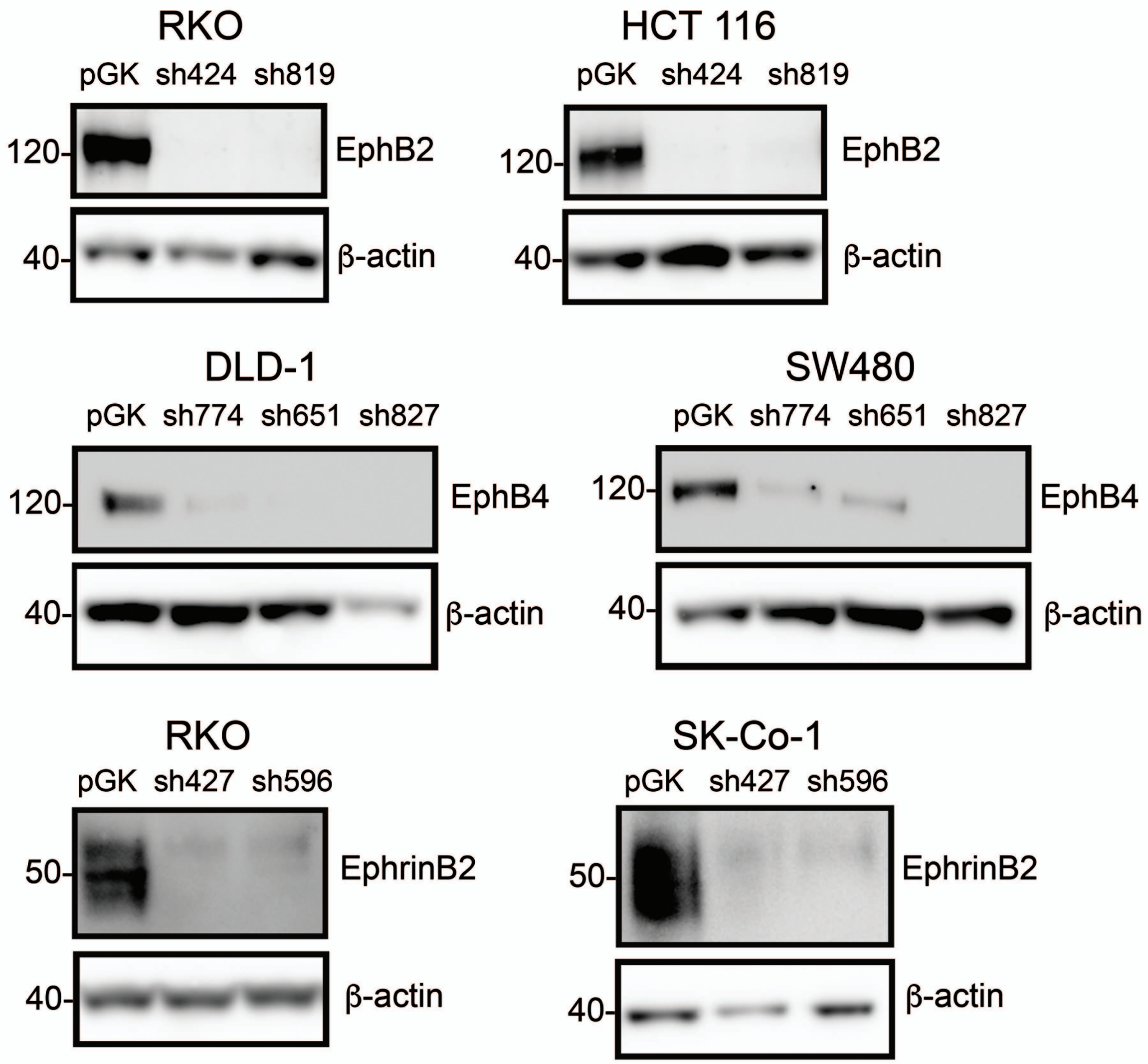

Fig. S3. EphrinB2, EphB2 and EphB4 silencing in colorectal carcinoma cell lines. Reduced EphrinB2, EphB2 and EphB4 protein levels in selected colorectal carcinoma cell lines 72 hours after shRNA infection. Representative immunoblotting results; β-actin reblotting used as a control.

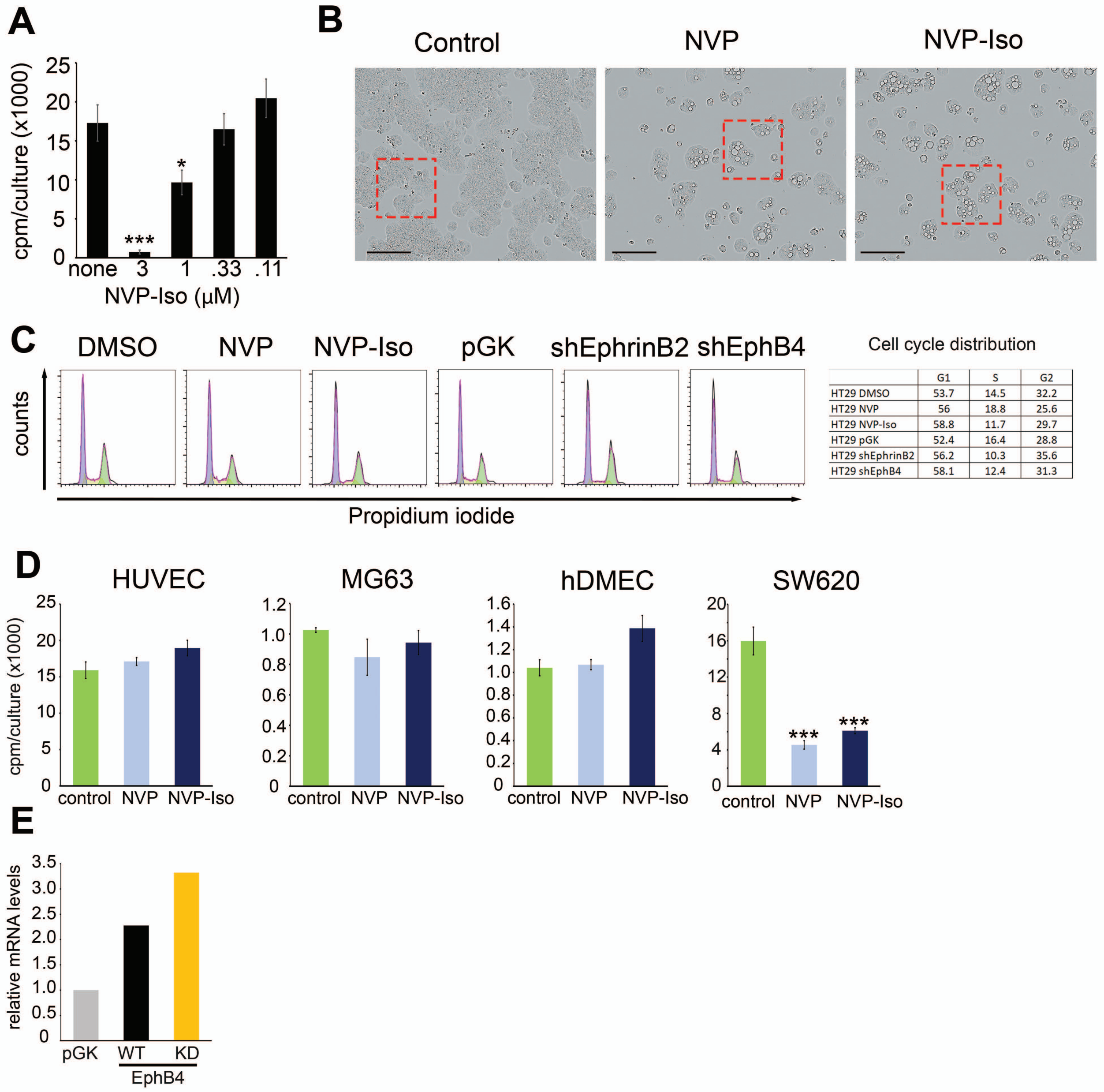

	G1	S	G2
HT29 DMSO	53.7	14.5	32.2
HT29 NVP	56	18.8	25.6
HT29 NVP-Iso	58.8	11.7	29.7
HT29 pGK	52.4	16.4	28.8
HT29 shEphrinB2	56.2	10.3	35.6
HT29 shEphB4	58.1	12.4	31.3

Fig. S4. (A)**,** NVP-Iso dose-dependently inhibits HT-29 cell proliferation. Results are expressed as mean cpm/triplicate cultures. (B)**,** Representative bright-field images showing reduced cell confluency in 3-day cultures supplemented with NVP or NVP-Iso (1µM). Boxed areas are magnified in main figure 4e; scale bars: 200µm. (C)**,** Cell cycle analysis in HT-29 cells treated with NVP or NVP-Iso (1µM) or transduced with pGK (control), shEphrinB2 (sh427), or shEphB4 (sh774) for 72hrs. Cells were fixed, permeabilized and stained with propidium iodide. (D)**,** Proliferation of HUVEC, MG63, hDMEC and SW620 cells after incubation with NVP or NVP-Iso (1µM) for 72hrs. Representative results of proliferation expressed as mean cpm/culture ±SD (triplicate cultures). (E)**,** Relative mRNA levels of EphB4 in HT-29 cells transduced with pGK control vector, WT EphB4 or kinase defficient (KD) mutant EphB4 (72hrs) by qRT-PCR. Statistical significance of difference calculated by two-tailed Student's t-test. $^*P<0.05$; $^{***}P\leq0.001$.

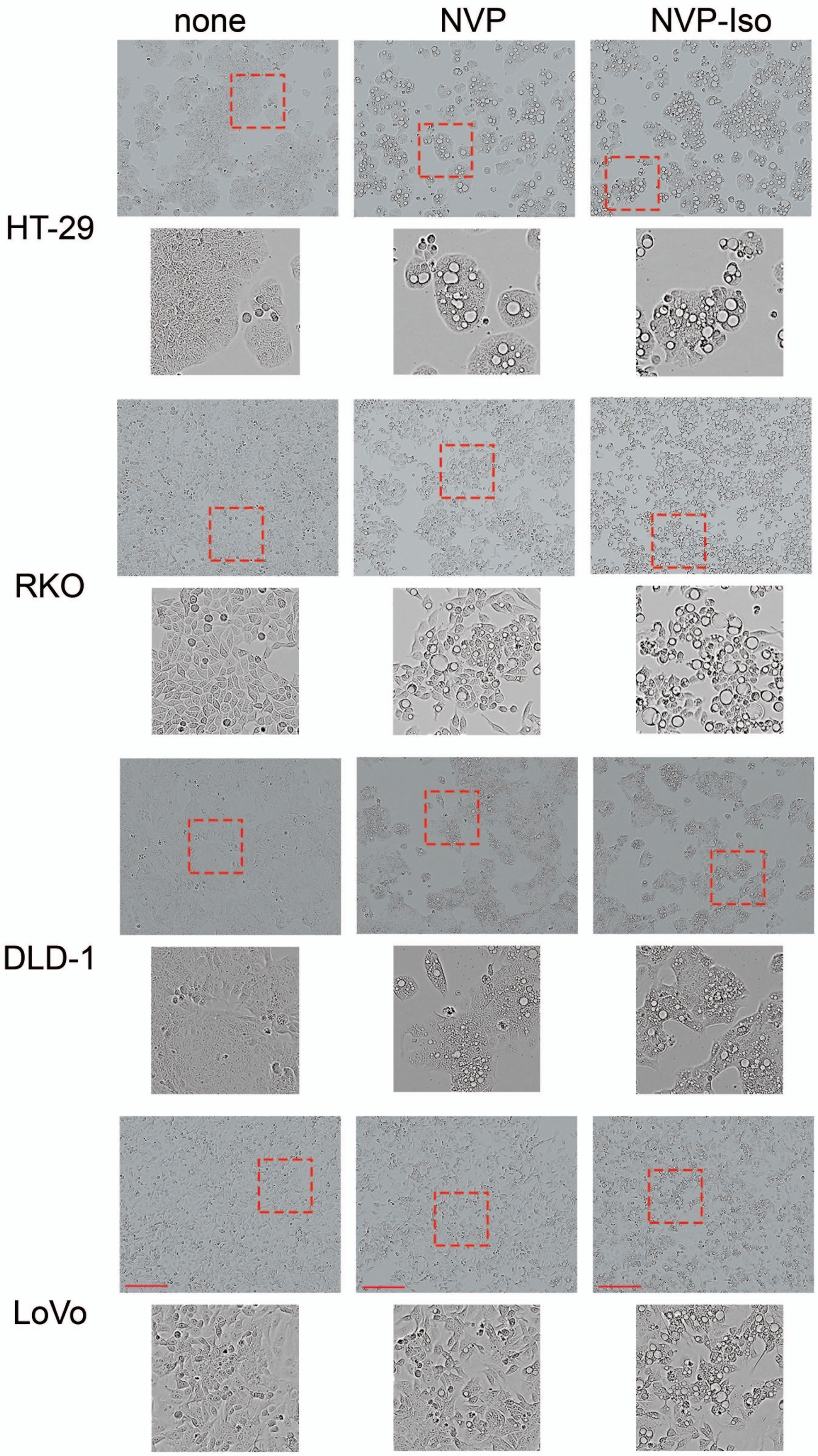

Fig. S5. Morphological change in colorectal carcinoma cell lines after incubation with NVP or NVP-Iso. Representative bright field Incucyte images of colorectal carcinoma cell lines incubated with 1μM NVP, 1μM NVP-Iso or none (control DMSO) for 48 hours. The areas limited by the red boxes are magnified immediately below. Scale bar: 200 μm.

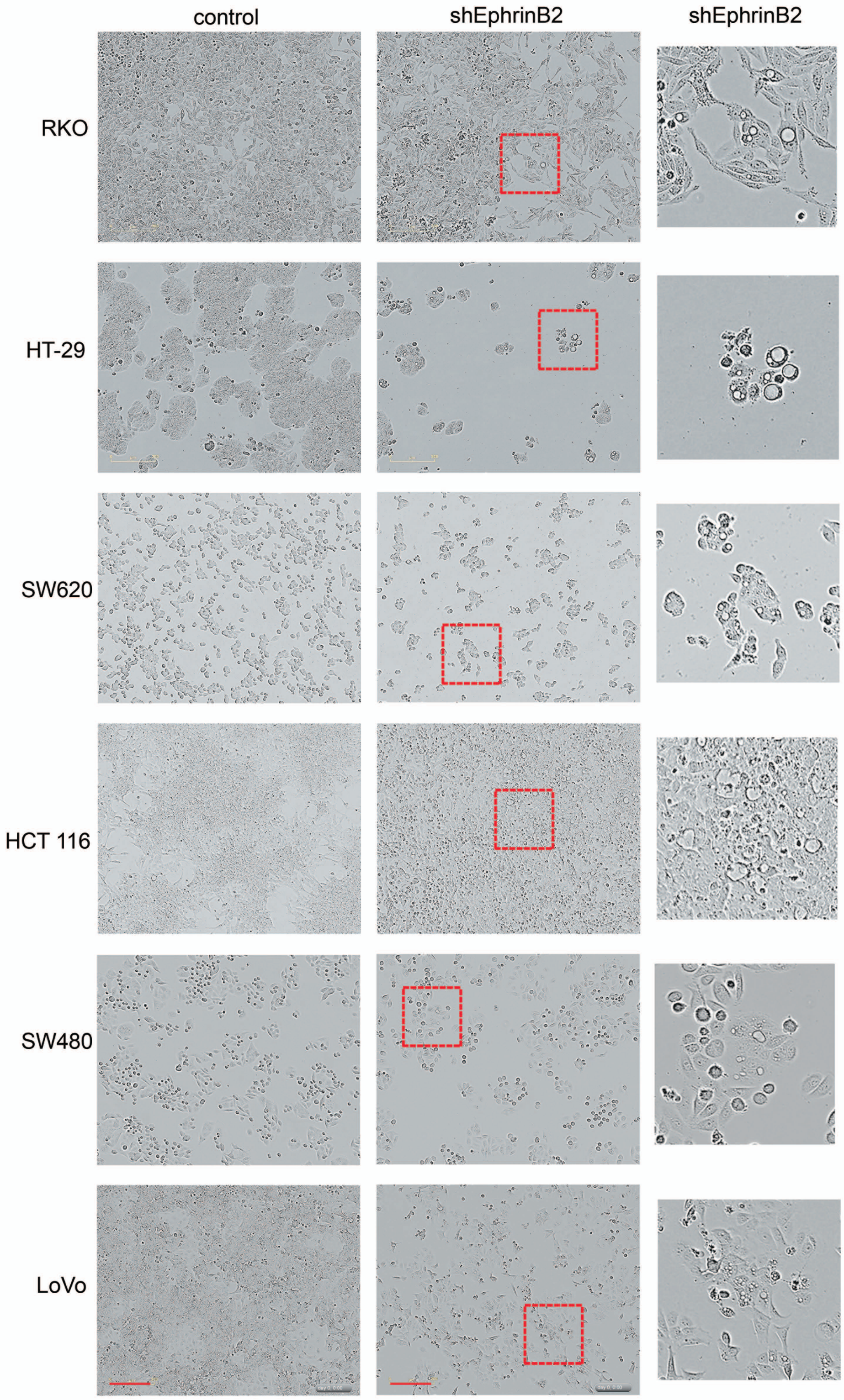

Fig. S6. Morphological change in colorectal carcinoma cell lines after EphrinB2 silencing. Representative bright field Incucyte images of colorectal carcinoma cell lines after EphrinB2 silencing with sh427 or infection with pGK control vector. Images obtained 48-120 hours after transduction. The areas limited by the red boxes are magnified on the right. Scale bar: 200 μm.

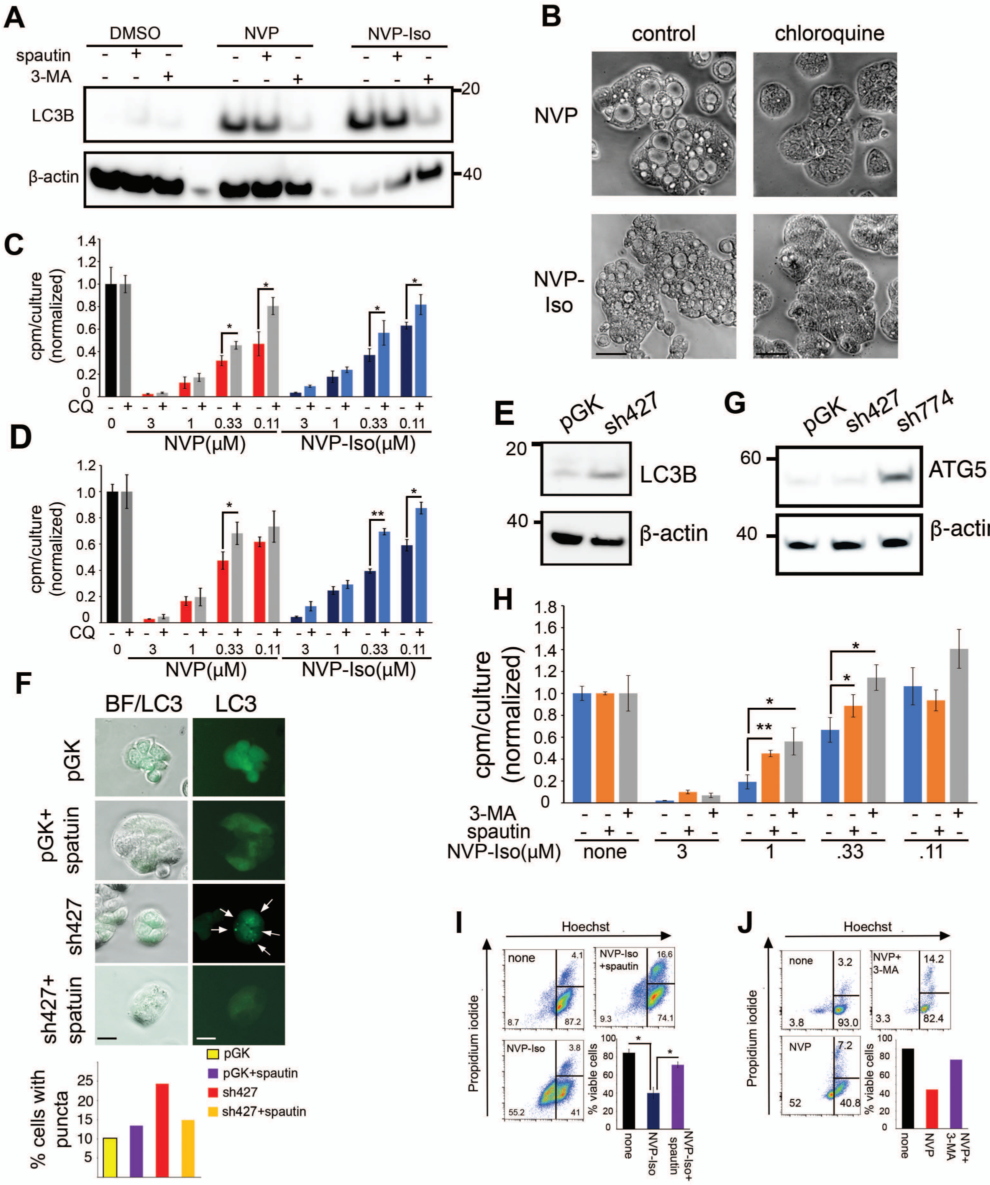

Fig. S7. Autophagy markers in colorectal carcinoma cells. (A) NVP and NVP-Iso (1μM) induce LC3B accumulation in SW620 cells after 72 hours; pre-treatment (4 hours) with spautin (5μM) or 3-MA (5mM) reduces this LC3B accumulation. (B) Chloroquine (10μM) reduces autophagic vacuoles in HT-29 cells treated with NVP or NVP-Iso (1μM); 72-hour culture; scale bars: 25μm. (C,D) Chloroquine (CQ; 10μM) enhances proliferation in HT-29 (C) and SW620 (D) colorectal cancer cells treated with NVP and NVP-Iso (0.11-3 μM); 72-hour culture; mean cpm/culture ±SD (triplicate cultures). (E) EphrinB2 silencing (sh427) induces LC3B accumulation in HT-29 cells. (F) EphrinB2 silencing induces autophagosomes in HT-29 cells; spautin (5μM) reduces this accumulation. Fluorescent “puncta” in LC3-expressing HT-29 cells; bright-field/fluorescent (left) and fluorescent (right) imaging; 20x objective. Bar graph: mean percent cells with ≥ 2 puncta/cell identified in 5 fields (400-500 total cells counted) in each experimental condition, scale bars: 10μm. (G) EphB4 silencing (sh774) induces the accumulation of the autophagy marker ATG5 in HT-29 cells. No ATG5 is induced after EphrinB2 silencing (sh427). (H) Pretreatment with spautin (5μM; 4 hours) and 3-MA (10mM) augments HT-29 cell proliferation inhibited by NVP-Iso (0.11-3 μM) at 72 hours; mean cpm/culture ±SD (triplicate cultures). (I) Spautin (5μM) and (J) 3-MA (5mM) reduce HT-29 cell death induced by NVP-Iso (0.5 μM) after 72 hours; representative profiles and quantitative results (bar graphs) of 3 independent experiments (I) or single (J) experiment (mean % ±SD). Statistical significance of group differences by two-tailed Student’s t-test. *P<0.05; **P≤0.01.

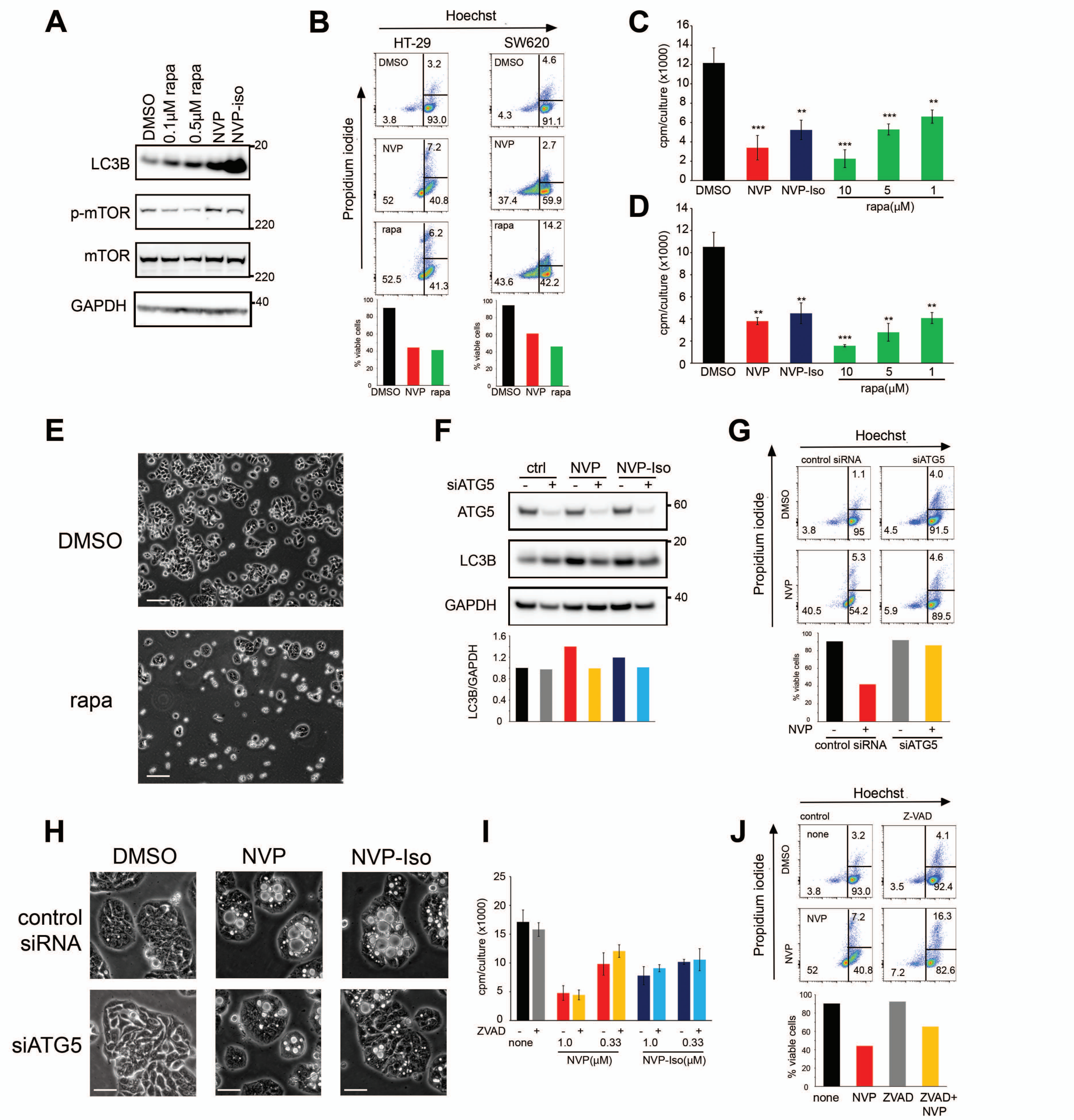

Fig. S8. Autophagy in colorectal carcinoma cells. (A) Rapamycin (rapa; 72-hour incubation) induces LC3B accumulation in HT-29 cells; immunoblots probed with specific antibodies to phospho(p)-mTOR (S2448), total m-TOR, LC3 and GAPDH. (B) Rapa (10μM) and NVP (1μM) induce similar cell death in HT-29 and SW620 cells after 72 hours; flow cytometry profiles (top) and quantitative results (bar graphs). (C, D) Rapa dose-dependently reduces cell proliferation in HT-29 (C) and SW620 (D) cells after 72 hours; mean cpm/culture ±SD (triplicate cultures). (E) Rapa (10 μM, 72-hour incubation) induces cell death in HT29 cells; representative bright field images; scale bars 50μm. (F) Effect of ATG5 silencing (24 hours prior to drug addition) on LC3B content in HT29 cells incubated for 72 hours with medium only or medium with NVP and NVP-Iso (1μM); immunoblots (top) and band quantification (bar graph). (G) Effect of ATG5 silencing (24 hours prior to drug addition) on HT-29 cell death induced by NVP (1μM) after 72 hours; flow cytometry profiles (top) and quantification (bar graph). (H) ATG5 silencing in HT-29 cells mitigates the autophagic morphology of HT-29 cells treated with NVP or NVP-Iso (1μM); scale bars: 12.5μm. (I) Z-VAD-FMK (ZVAD; 0.5μM) minimally affects the proliferation of HT-29 cells incubated for 72 hours with NVP or NVP-Iso (0.33 or 1 μM); mean cpm/culture ±SD (triplicate cultures). Statistical significance of group differences by two-tailed Student's t-test. **P≤0.01, **P≤0.001.
